# Neuroprotective Effects of Carnosine Against Corticosterone‐Induced Depression and Memory Impairment

**DOI:** 10.1002/fsn3.71837

**Published:** 2026-05-03

**Authors:** Muneezah Elahi, Noreen Samad, Ali Irfan, Natasha Manzoor, Muhammad Zeeshan Ahmed, Emilio Mateev, Yousef A. Bin Jardan

**Affiliations:** ^1^ Department of Biochemistry, Faculty of Science Bahauddin Zakariya University Multan Pakistan; ^2^ The Green Institute of Chemical Biomedical and Environmental Sciences (GICBES) Lahore Pakistan; ^3^ Department of Pharmaceutical Chemistry, Faculty of Pharmacy Medical University – Sofia Sofia Bulgaria; ^4^ Department of Pharmaceutics, College of Pharmacy King Saud University Riyadh Saudi Arabia

**Keywords:** antioxidant potential, carnosine, cognition, corticosterone, depression, serotonin

## Abstract

Corticosterone (Crt) is reported to induce oxidative stress in experimental animals. Carnosine (Crn), a well‐known dipeptide antioxidant synthesized endogenously from β‐alanine and L‐histidine, neutralizes oxidative stress. The current study examines the influences of Crn on Crt‐induced behavioral alterations (depressive‐like behaviors and cognitive performance), oxidative damage, antioxidant enzymes activity, inflammatory markers, serotonin metabolism, and histopathology in female rats. Thirty‐six animals were allocated to six groups (*n* = 6): (i) vehicle (vh) + vh (1 mg/kg) (ii) vh + Crn (20 mg/kg), (iii) vh + Crn (50 mg/kg), (iv) vh + Crt (20 mg/kg) (v) Crt + Crn (20 mg/kg), and (vi) Crt + Crn (50 mg/kg). All respective doses were given intraperitoneally (i.p) for 2 weeks. After treatment, behavioral testing was done via the Tail suspension test (TST) for depressive‐like behavior and the Morris Water Maze (MWM) for spatial memory. Crn treatment had significant effects on Crt‐induced behavioral impairments, such as depressive‐like behavior and cognitive dysfunction. After completing behavioral tests, the rats underwent decapitation, and the hippocampus was separated for further biochemical and neurochemical analyses. Outcomes revealed that Crn mitigates depressive‐like behaviors and cognitive dysfunction. Crn reduces oxidative stress and inflammatory cytokine levels while improving antioxidant enzyme activity, restoring cholinergic and serotonergic transmission, and brain (hippocampus) morphology following Crt‐administration. The in silico analyses also demonstrate its strong binding affinity with monoamine oxidases (MAO) A and B, with an energy of −7.1 and −6.7 kcal/mol, respectively. In conclusion, the present results showed that Crn possesses strong antioxidant properties and reduced Crt‐induced depressive‐like behavior and memory impairment due to its effective abilities as antioxidant and neuromodulator. Supplementation with Crn as a dietary component may provide protective benefits against Crt‐induced depression‐like behavior and memory impairment.

## Graphical abstract

This main findings of the study and illustrates the key workflow.

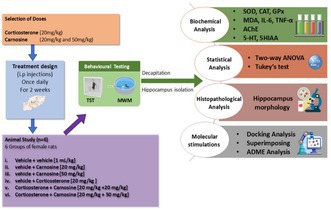

## 1 Introduction

Oxidative stress (OS) is an inequality between the overproduction of reactive oxygen species (ROS) and the insufficient availability of antioxidants or radical‐scavenging processes (Dash et al. 2025). Excess ROS can alter the structural integrity of biomolecules and initiate signaling pathways related to neurological pathologies (Chatterjee 2016). ROS cause lipid peroxidation, which forms reactive aldehydes that interfere with membrane integrity (Endale et al. 2023). These cause oxidation of proteins and carbonylation, which disrupts structure and enzyme activity (Stadtman and Berlett 1998). ROS also leads to DNA base lesions and strand breaks, which encourage mutagenesis and genomic instability (Srinivas et al. 2019). Additionally, ROS are known to be critical mediators of neurodegenerative diseases, that is, Parkinson's disorder (Atsushi and Tamano 2020) Alzheimer's disease (Birla et al. 2020) anxiety and depression (Grases et al. 2014) and memory impairment (Thingore et al. 2021). Mitochondrial dysfunction is a key component in dementia and/or cognitive impairment due to the pathological interaction of mechanisms that affect neuronal function through ATP impairment, enhancement of ROS, and activation of apoptotic pathways, thus affecting synaptic function and plasticity (Lin and Beal 2006; Swerdlow 2018). Neuroinflammation, driven by microglial activation and pro‐inflammatory cytokines, further contributes to synaptic dysfunction, neuronal injury and cognitive dysfunction (Heneka et al. 2015; Zhang et al. 2025). Simultaneously, the degeneration of cholinergic neuron, not only reduced acetylcholine (ACh) function but also contribute in the cognitive deficits and indicative of a role of neuroinflammation and cholinergic damage in dementia and/or memory impairment (Chen et al. 2022).

Repeated stress activates the hypothalamic–pituitary–adrenal (HPA) axis, thereby enhancing secretion of glucocorticoids (GC), that is, cortisol in humans and corticosterone (Crt) in rodents (McEwen 2007). Prolonged exposure to these GCs, such as due to Crt‐administration, serves as an important mechanistic pathway in which hyperactivation of the HPA axis (Huang et al. 2011) produces neurotoxicity, hence impairing normal hippocampus functions, followed by depressive behavior and memory decline (Pérez‐Nievas et al. 2007) In the brain, structural changes in response to Crt‐induced stress have been well described, especially in the hippocampus (McEwen 2007). Crt exposure reduces prefrontal and hippocampal dendrites (Radley et al. 2006) while hyperactivating amygdala circuits following depression symptoms and cognitive deficits (Vyas et al. 2006). Crt triggers necrotic cell death by inhibiting antioxidant defenses and increasing free radical generation (Zhao et al. 2008). The brain's high oxygen demand, lipid content, and limited antioxidant defenses mark oxidative stress, a key driver of neurodegeneration and behavioral alteration, that is, memory alteration and depression (Bhatt et al. 2020; Tanti and Belzung 2010). Crt also causes alterations in neuronal integrity, neurotransmitter regulation, eventually leading to cognitive impairments (Lim et al. 2020). Elevated Crt is also known to increase levels of inflammatory markers, including IL‐6 (interleukin‐6) and TNF‐α (tumor necrosis‐factor‐α) in the animal model (Kelly et al. 2018), leading to impaired cognition and mood (Yu et al. 2022). The model of chronic Crt treatment is a commonly used model of cognitive impairment and depression because it recapitulates several pathological processes involved in the neurodegenerative and mood disorders, which are not limited to oxidative stress. For instance, the model of chronic GC treatment was used to show the induction of mitochondrial dysfunction through impaired bioenergetics and activation of apoptotic pathways (Du et al. 2009; Shaw et al. 2024), the induction of neuroinflammatory processes through microglial priming and increased production of pro‐inflammatory cytokines (Frank et al. 2011; Munhoz et al. 2006), and the disruption of neurotransmitter systems such as glutamatergic, GABAergic, and monoaminergic neurotransmission, thereby impairing synaptic plasticity, mood regulation, and cognition (Duman and Aghajanian 2012; Mahar et al. 2014). These changes are very similar to those seen with depression and dementia, including hippocampal atrophy and synaptic dysfunction (Krishnan and Nestler 2008). Overall, these data support Crt‐induced cognitive decline and depression‐like neuropathology as multifaceted and mechanistically relevant models of stress‐induced cognitive decline and depression.

However, various amino acids have been reported to mitigate oxidative stress, thereby improving behavioral and cognitive deficits (Sánchez‐Martínez et al. 2020). Histidine‐containing dipeptides like carnosine (Crn), anserine, and homocarnosine are gradually being regarded as antioxidative and neuroprotective in brain areas that are vulnerable to depression (Kabthymer et al. 2025) and memory impairment (Masuoka et al. 2019; O'Toole et al. 2025).

Crn (C_9_H_14_N_4_O_3_), a nitrogen‐containing dipeptide, is a combination of L‐histidine and β‐alanine (Everaert et al. 2013), localized in skeletal muscle and various excitable tissues (Boldyrev et al. 2013). Crn exerts its main effect via its antioxidant properties by decreasing intracellular levels of ROS and modulation of the endogenous antioxidant status (Caruso et al. 2017). The antioxidant capacity of Crn is due to its imidazole ring, which rapidly reacts with ROS, forming an imidazole complex, thus limiting its oxidative activity in a highly efficient manner (Boldyrev et al. 2013). The Blood–Brain Barrier (BBB) permeability of Crn via amino acid transporters highlights its potential as a therapeutic agent for various brain dysfunctions, that is, Alzheimer's disorder and Parkinson's disease (Schön et al. 2019). It is reported for its anti‐glycating and anti‐aggregative activity, anti‐inflammatory action, and antioxidant activity in various animal models. It also implies that Crn is involved in maintaining brain health and cognitive performance (Meftahi and Jahromi 2023). Findings have shown that Crn plays a significant role in serotonin metabolism in various regions (Banerjee and Poddar 2016). Crn also acts as an inhibitor of monoamine‐oxidase A, an enzyme that degrades 5‐HT, thereby enhancing synaptic serotonin availability. This regulation of serotonergic neurotransmission is the key aspect of Crn's mechanism of action (Banerjee and Poddar 2015). In addition, Crn is also reported to regulate the cholinergic system of the brain (Ishioh et al. 2025).

Crn was selected for this study due to its unique dual properties as both a potent antioxidant and a neuromodulator, with demonstrated effects on acetylcholinesterase (AChE) inhibition, serotonergic modulation, and neuroprotection in preclinical models. Unlike many other antioxidants, Crn can cross the BBB (Schön et al. 2019) and directly interact with neuronal targets (Zhang et al. 2015), making it particularly suitable for investigating cognitive and behavioral outcomes. Pre‐clinical studies also have confirmed that systemic administration of Crn in rodent models at doses of 10, 20, and 50 mg/kg/day has been demonstrated to be effective in enhancing functional recovery and reducing oxidative stress and inflammation. These doses are within a previously validated dosage range for the administration of this drug in vivo (Guliaeva et al. 1989; Mirzakhani et al. 2018; Ommati et al. 2020; Taskin 2014). Based on this background, it is hypothesized that Crn at doses of 20 and 50 mg/kg (used in the present study) may mitigate Crt‐induced depression and cognitive dysfunction as assessed through behavioral, biochemical, neurochemical, histopathological, and in silico studies; while previous studies have suggested the neuroprotective potential of Crn, it is proposed that combining these multiple approaches in a Crt‐induced model could provide a more comprehensive understanding of its potential protective mechanisms.

## 2 Experimental Approach

### 2.1 Reagents and Chemicals Used

L‐Crn was acquired from NOW Foods (Bloomingdale, IL, USA) in capsule form (500 mg per capsule). Crt, 2‐Thiobarbuturic acid (TBA), Trichloroacetic acid/TCA (CCL_3_COOH), Disodium carbonate (Na_2_CO_3_), Nitro BT, that is, Nitro blue tetrazolium (C_40_H_30_C_l2_N_10_O_6_), hydroxylammonium chloride (NH_2_OH·HCl), Dipotassium dichromate, glacial acetic acid, L‐glutathione (reduced form), Acetylthiocholine Iodide (C_7_H_16_INOS), Ellman's reagent, that is, DTNB (dithio‐bis nitrobenzoic acid), sodium phosphate dibasic (Na_2_HPO_4_) were procured from Sigma‐Aldrich Inc. (St. Louis, USA). While hydrogen peroxide (H_2_O_2_), sodium carbonate, Sodium trinitride (i.e., sodium azide or NaN_3_), Ethylenedinitrilo tetra acetic acid, that is, EDTA (C_10_H_16_N_2_O_8_) were obtained from British Drug House (BDH, Dorset, UK).

### 2.2 Animals

For this study, 36 locally bred Sprague–Dawley female albino rats (180–200 g; 6–8 weeks old) were procured from the University of Lahore, Lahore‐Pakistan. In this study, female rats were selected because sex differences can influence neurobehavioral and neurochemical responses. It has been suggested that female rodents have unique behavioral profiles and stress responses in relation to their male counterparts, which may have an impact on the results of depression and cognitive dysfunction models (Becker et al. 2016). The animals were kept in standard polypropylene cages. The bedding material used was sterilized wood shavings. The cages were kept in a controlled environment with a temperature range of 23°C ± 3°C, relative humidity of 50% ± 10%, and a 12‐h light–dark cycle with lights on at 07:00 h. The animals had access to food and water ad libitum. The food used was standard rodent diet (Bocarsly et al. 2012) that was prepared in the laboratory. All the food and water used in the experiment was checked for any contaminants. The animals had a 1‐week acclimatization period before the experimental procedures began to adjust to the new environment. Strict adherence to all ethical guidelines was maintained, and the research protocol was sanctioned by the Departmental Bioethical Committee (Biochem/2025/D‐2010; Dated: 14.04.2025). Animals were randomly assigned to experimental groups to ensure unbiased group allocation. It is stated that the research work was designed and reported in accordance with the ARRIVE guidelines.

### 2.3 Experimental Protocol

All experiments conducted on adult female rats. Therefore, sex distribution across the experimental group was uniform. Thirty‐six female rats were randomly assigned to six groups (*n* = 6 rats in each group) as reported in previous studies (Samad et al. 2019).

- vh + vh (1 mL/kg),
- vh + Crn [20 mg/kg (Mirzakhani et al. 2018)],
- vh + Crn [50 mg/kg (Sun et al. 2017)],
- vh + Crt [20 mg/kg (Li et al. 2019)],
- Crt + Crn (20 mg/kg + 20 mg/kg),
- Crt + Crn (20 mg/kg + 50 mg/kg).

**FIGURE 1:**
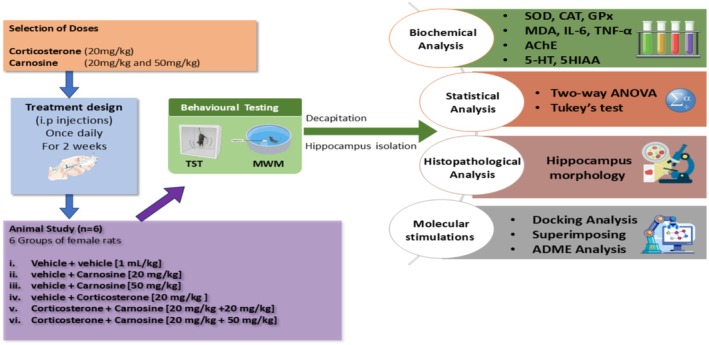
Study workflow.

Crt and Crn were dissolved in saline and injected into their respective groups sequentially at 3–5 min interval respectively for 14 days. Vehicle (saline, 1 mL/kg; intraperitoneally, i.p) was given to the control group, as done earlier by Mirzakhani et al. (2018). Behavioral tests, the Tail Suspension Test (TST) on the 15th day to determine depression related behaviors, and Morris Water Maze (MWM) on the 16th day to determine memory dysfunction. Acquisition and short‐term memory (STM) were determined on day 16, while long‐term memory (LTM) was determined on day 17 with assessments carried out by blind observers. Rats were decapitated 1‐h after the behavior analysis. The decapitation was performed under profound general anesthesia induced with 4% isoflurane delivered in an induction chamber (Shakeel et al. 2020) and brain tissues were harvested as reported by Tabassum et al. (2017). The hippocampal samples were separated (Samad et al. 2022) and kept at −80°C for later biochemical, neurochemical, and histopathological examinations (Figure 1).

### 2.4 Behavioral Estimations

#### 2.4.1 Tail Suspension Test

TST is a commonly employed behavioral assay for assessing depressive‐like behaviors in rodents (Castagné et al. 2010) (Brandão et al. 2023). The test was performed by using a rectangular plastic box (55 cm height 60 cm width 11.5 cm depth). The apparatus was designed to assess immobility in animals as an indicator of depressive‐like behaviors. Rats were suspended individually by their tails from an aluminum bar, maintaining a height of 20–25 cm above the floor to prevent contact with the box walls. An investigator who was blinded to the experimental conditions assessed all the activity manually. Each animal was observed for 5 min to assess the degree of immobility, indicative of depression‐like behaviors.

#### 2.4.2 Morris Water Maze Test

The Morris water maze (MWM) test was carried out to assess cognitive function in rats (Haider et al. 2012, 2007). A circular pool filled with non‐transparent water and a hidden wooden platform was used for the MWM test. The memory and cognitive function of rats were assessed based on their ability to find a hidden platform. The Morris water maze test was divided into two parts: the training and evaluation phases. Initially, the rats were trained to find a hidden platform in a pool of clean water. The platform was submerged in water at a depth of 2 cm and was marked with a pole. The rats had a time limit of 2 min to find the platform and step onto it. The time taken for the rat to find the platform and step onto it was referred to as the rat's escape latency and was an important factor in the Morris water maze test. After 4 h of training in the water maze, the rats were given a short‐term memory test. The water in the pool was made opaque by adding a white poster. The rats had a time limit of 2 min to find the platform. After the short‐term memory test, the rats were assessed for long term memory test after 24 h. Throughout these phases, the platform was waterlogged 2 cm lower than the surface of water, and the rats were given the task to re‐locate the platform within 2 min. The procedure was followed by the blind observer, manually for 2 min.

### 2.5 Biochemical Estimations

Hippocampal tissue was rinsed with 0.9% NaCl and homogenized. The sample (10%) was homogenized using pH 7.4 phosphate buffer (0.1 M) and then centrifuged (10,000 × g, 10 min, 4°C), and the supernatant layer was used in further evaluation.

#### 2.5.1 MDA

In the current study, oxidative stress was determined by calculating malondialdehyde (MDA) content in brain tissue. For the estimation of MDA, the mixture was attained by adding TCA (2 mL) and TBA (2 mL) reagents to tissue homogenate (3 mL). Then we boiled the mixture for 15 min, cooled it down to room temperature, and centrifuged (35,000 rpm) for 10 min. The resulting light‐pink supernatant was harvested, and the absorbance at 532 nm was recorded as previously explained by (Chow and Tappel 1972). The contents of MDA were illustrated as mM of MDA/gm of brain tissues.

#### 2.5.2 SOD

Superoxide dismutase (SOD) activity was estimated according to the procedure of (Naskar et al. 2009). The reaction mixture included 0.5 mL of tissue homogenate, NaHCO_3_ (0.1 mL), Nitro blue tetrazolium (C_40_H_30_C_12_N_10_O_6_; 0.4 mL), and EDTA (0.2 mL). 0.4 mL of NH_2_OH·HCl was added to start the reaction. The spectrophotometer analysis was performed at 570 nm. Activity of SOD was shown as % inhibition.

#### 2.5.3 CAT

Catalase (CAT) activity was assessed according to the procedure described by (Pari and Latha 2004). The experimental mixture included 1 mL of pH 7.4 phosphate buffer, a homogenate of tissue (0.1 mL), and 0.4 mL of H_2_O_2_. The enzyme activity was determined at 570 nm spectrophotometrically. The activity of CAT in tissue sample was expressed as μmoL of H_2_O_2_ consumed/min/mg of protein.

#### 2.5.4 GPx

The activity of GPx in hippocampus tissue was assessed, following the methods of (Pari and Latha 2004) and Flohe (Flohé and Günzler 1984). The reaction mixture included 0.1 mL Sodium trinitride (NaN_3_), 0.1 mL H_2_O_2_, 0.2 mL educed glutathione (GSH), 0.3 mL of pH 7.0 phosphate buffer, and 0.2 mL supernatant of the brain. This mixture was maintained at 37°C to complete the reaction for 15 min, followed by termination of the reaction using trichloroacetic acid (TCA, 0.5 mL). The mixture was centrifuged at 35,000 rpm for 5 min, and 0.1 mL of the resultant supernatant was added to Na_2_HPO_4_ (0.2 mL) and DTNB (0.7 mL). Absorbance was read at 420 nm. Activity was illustrated as μmoL of residual GSH/min/mg of protein.

#### 2.5.5 AChE

Activity of AChE was measured based on (Ellman et al. 1961), where acetylthiocholine iodide (ATC) was used as a substrate. A 0.4 mL aliquot of homogenate of hippocampus was added to an incubation mixture consisting of 2.6 mL of pH 8.0 phosphate buffer (0.1 M), and Ellman's reagent (100 μL). The mixture was aerated, and then we measured absorbance at 412 nm after the reaction stabilized, giving a baseline reading. Then 5.2 μL of acetylthiocholine iodide was added to introduce enzymatic activity. Absorbance was measured again at 412 nm at zero minutes and at 10 min (25°C). The enzymatic activity of AChE was quantified and expressed as μmoL/min/g of brain tissue.

### 2.6 Inflammatory Markers

The inflammatory cytokines, that is, IL‐6 (Abcam; Cat#ab234570; range 125–8000 pg/mL) and TNF‐α (Cat#ab100784; range 82.3–20,000 pg/mL) were quantified by using the ELISA method for the hippocampus region, as earlier described. (Samad et al. 2021).

### 2.7 Neurochemical Estimation

#### 2.7.1 Serotonin Metabolism

The 5‐HT and 5‐HIAA levels were quantified using a previously described method (Haider and Tabassum 2018). Frozen hippocampal tissue samples were homogenized with an electric homogenizer in a solution containing 0.4 M PCA, 0.1% sodium metabisulfate, 0.1% EDTA, and 0.01% cysteine. Then these samples were refrigerated for 15 min and centrifuged for 15 min at 10,000 rpm and 4°C to facilitate the protein preparation. The resulting supernatant was analyzed to evaluate serotonin levels in the hippocampus. The homogenized brain tissue was used to analyze the levels of 5‐HT and 5‐HIAA using reversed‐phase HPLC with an electrochemical detector (Shimadzu LEC 6A) set at +0.8 V. A 20‐μL aliquot of each sample was injected onto a 5‐μm particle size Shim‐pack ODS column (4.0 mm inner diameter, 150 mm length). The mobile phase, which was a 0.1 M phosphate buffer at a pH of 2.9 containing 0.023% octyl sodium sulfate, was used at a flow rate of 1.0 mL/min under a pressure of 2000–3000 psi. Peaks were identified using standard reference compounds.

### 2.8 Histopathological Analysis

A 2–5 μm‐thick section of the formalin‐fixed brains was taken out for histopathological analysis by a blinded observer, and the resulting paraffin was preserved for microscopic examination. As previously stated, slides were inspected under a 200× light microscope after being stained with hematoxylin (H) and eosin (E) (Suvarna et al. 2018).

### 2.9 In Silico Analysis

#### 2.9.1 Computational Docking

To evaluate the binding affinity of Crn with MAO‐A and MAO‐B, we performed molecular docking analysis. The 3D structures of MAO‐A and MAO‐B were acquired from the Protein Data Bank (PDB) (https://www.rcsb.org/, accessed November 11, 2025) using PDB IDs 2Z5Y and 2XFU, respectively. For the docking, we prepared the protein structures by removing ligands, water molecules, heteroatoms, and side chains using Discovery Studio 2025. Then, we repaired the missing atoms, added polar hydrogens, and assigned Kollman charges using MGL tools. After that, the grid box for the whole molecule was set for docking analysis. Similarly, the Crn molecule was accessed from PubChem (https://pubchem.ncbi.nlm.nih.gov/) with CID: 439224. The energy minimization and Kollman charges of Crn were set in MGL tools. The computational docking was performed using PyRx v.0.8 under the AutoDock Vina algorithm. The best binding showing complexes was visualized using PyMol and Discovery Studio Visualizer (Naskar et al. 2009).

#### 2.9.2 High Structural Similarity Between Human and Rat MAO‐A Bound With Crn

To evaluate the similar effect of Crn in humans and rats, we compared the docking results of human and rat MAO‐A (i.e., 2XFU and 1O5W, respectively) with Crn using a structural superimposition technique by considering near‐identical overall folds and a conserved active site architecture in enzymes of both species. After following the docking procedure as mentioned above, we superimposed the human MAO‐A complex over the rat MAO‐B complex and calculated the RMSD value of both ligands.

### 2.10 Swiss ADME

Insights into the physicochemical and pharmacokinetic attributes of Crn serve as the basis for the design of antidepressant compounds. The Swiss ADME server was utilized to analyze the ADME toxicity of Crn.

### 2.11 Statistical Analysis

All the values are presented as mean ± SD, and statistical assessments were conducted by two‐way Anova with Tukey's post hoc test using SPSS software (version 21). A *p* value of < 0.05 was considered statistically significant.

## 3 Results

### 3.1 Tail Suspension

**FIGURE 2:**
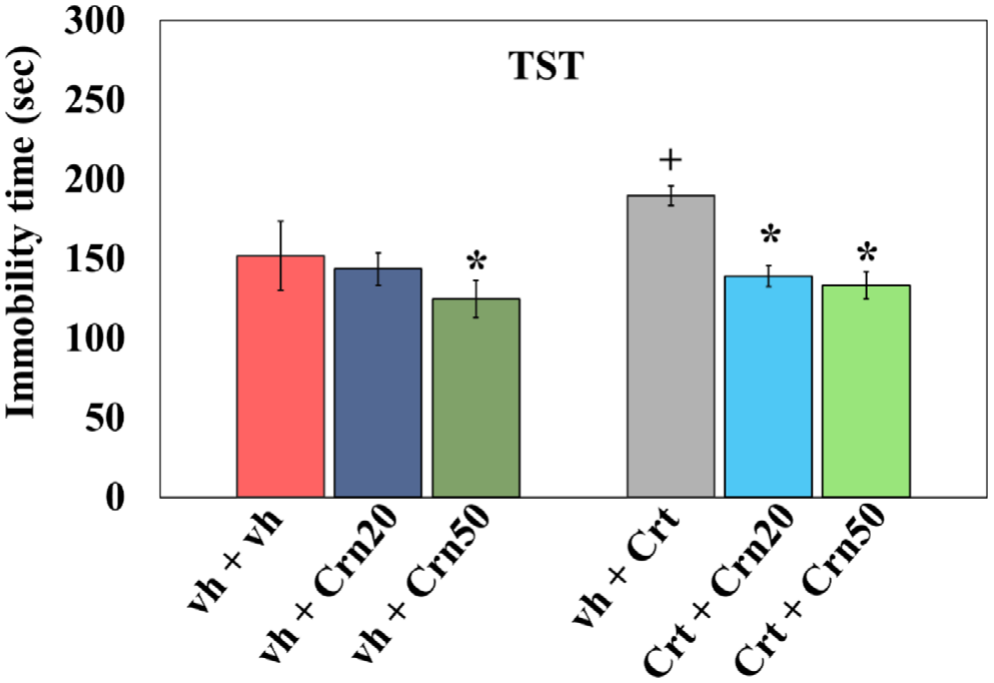
Result of Crn on immobility duration in TST in vh‐, Crn‐, and Crt‐administered rats. All data are expressed as ± SD (*n* = 6). Data were statistically analyzed through two‐way Anova with Tukey's post hoc comparisons, that is, *p < 0.05 when compared with vh + vh group; +*p* < 0.05 when compared with vh + Crt group.

The Figure 2 illustrates the effect of Crn on Crt‐injected depressive‐like behaviors, as assessed by TST. 2‐way Anova revealed notable influence of Crn {(*F*_2,30_ = 35.11, *p* = 0.000), Crt (*F*_1,30_ = 11.43, *p* = 0.002), and interaction Crn × Crt (*F*_2,30_ = 8.58, *p* = 0.001)}. Tuckey's post hoc analysis exhibited that Crt treatment resulted in prolonged immobility. Administration of Crn at both doses produced a decline in the immobility phase in vh and Crt‐treated rats.

### 3.2 Morris Water Maze Test

**FIGURE 3:**
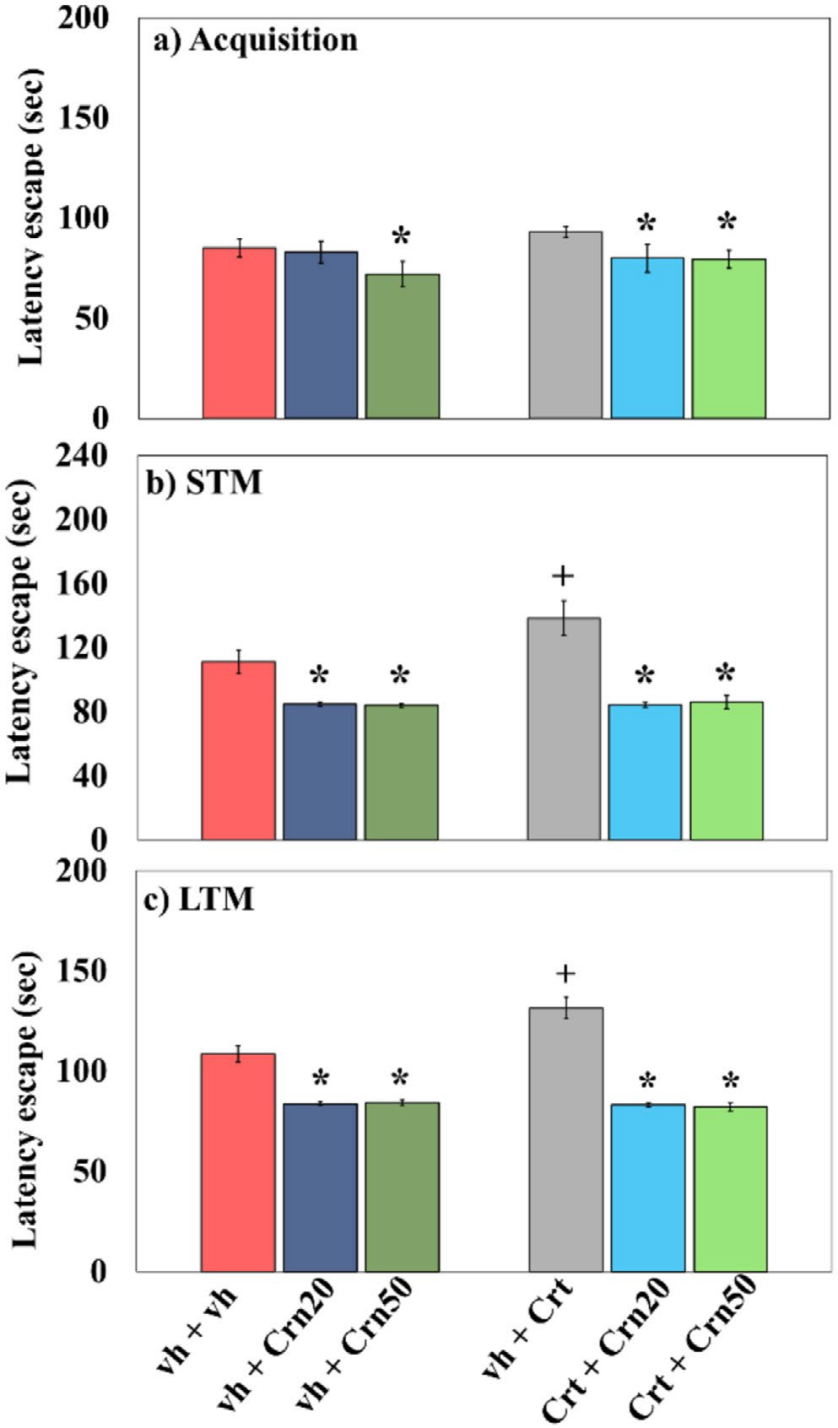
Influence of Crn evaluated by MWM in vh and Crt‐administered rats. Values are mean ± SD (*n* = 6). Analysis of data (two‐way Anova) followed by Tukey's test was used for statistical evaluation. The level of significance is represented as *p < 0.05 relative to vh + vh‐treated animals and + *p* < 0.05 relative to vh + Crt‐treated animals.

Figure 3 displays the influence of Crn on performance in memory tasks in Crt and vh‐treated rats observed in this test. Latency period to escape was evaluated across three consecutive sessions, that is, training (acquisition), short‐term (1‐h), and long‐term (24‐h) memory assessments. All the data were subjected to a two‐way Anova.

The acquisition data demonstrated significant effects of Crn {(*F*_2,30_ = 20.3; *p* = 0.000) and interrelation Crn × Crt (*F*_2,30_ = 4.5; *p* = 0.019) while insignificant effects of Crt (*F*_1,30_ = 3.6; *p* = 0.067)}. Tukey's multiple comparison test displayed that Crt administration prolonged latency to escape in the acquisition period. Contrary wise, both doses of Crn reduced latency escape in vh + Crt and Crt + Crn‐treated rats.

The findings related to STM data demonstrated a pronounced effect of Crn {(*F*_2,30_ = 203.8; *p* = 0.000), Crt (*F*_1,30_ = 25.5; *p* = 0.000), and combination Crn × Crt (*F*_2,30_ = 22.2, *p* = 0.000)}. Tukey's test displayed that Crt administration prolonged latency escape during the valuation of short‐term memory. Contrarily, both doses of Crn reduced latency escape in Crt and vh‐treated rats.

LTM data showed a considerable result of Crn {(*F*_2,30_ = 577.6; *p* = 0.000), Crt (*F*_1,30_ = 75.3; *p* = 0.0001) and interrelation Crn × Crt (*F*_2,30_ = 72.7; *p* = 0.000)}. Statistical analysis through Tukey's test exhibited that Crt treatment extended latency escape. Conversely, both doses of Crn reduced latency escape in vh + Crt and Crt + Crn‐treated rats.

### 3.3 MDA

**FIGURE 4:**
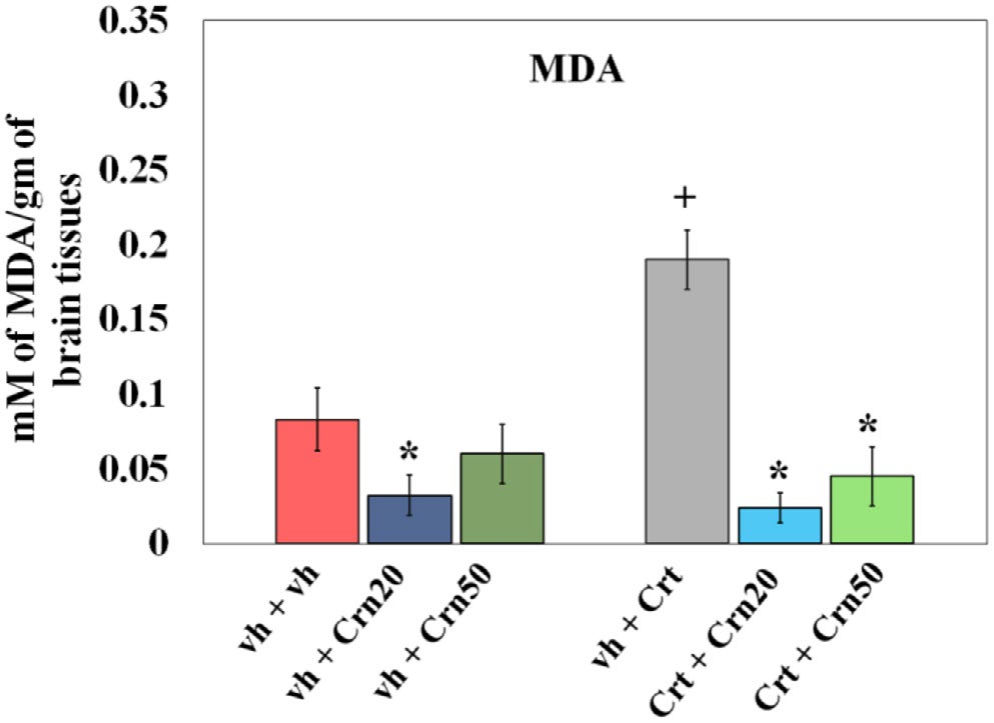
Effects of Crn treatment on MDA content in vh and Crt administered rats. The data set was investigated by two‐way Anova, followed by Tukey's test for multiple comparisons. Values are mean ± SD (*n* = 6). Statistical difference is represented as *p < 0.05 versus relative to vh + vh group and + *p* < 0.05 relative to responding control vh + Crt administered animals. Administration of both doses of Crn resulted in suppressed MDA levels in vh and Crt‐administered groups.

Effects of Crn on MDA levels in the rat hippocampus are shown in Figure 4. Results obtained through 2‐way ANOVA exhibited a strong response pattern of Crn {(*F*_2,30_ = 88.527, *p* = 0.000), Crt (*F*_1,30_ = 16.317; *p* = 0.000), and combination Crn × Crt (*F*_2,30_ = 33.314; *p* = 0.000)}. Tukey's test exhibited that Crt administration elevated oxidative stress through an increase in levels of MDA when compared to vh‐treated rats. Administration of Crn (20 and 50 mg/kg) reduced MDA levels in the hippocampal tissue of vh + Crt and Crt + Crn‐treated rats.

### 3.4 Inflammatory Markers

**FIGURE 5:**
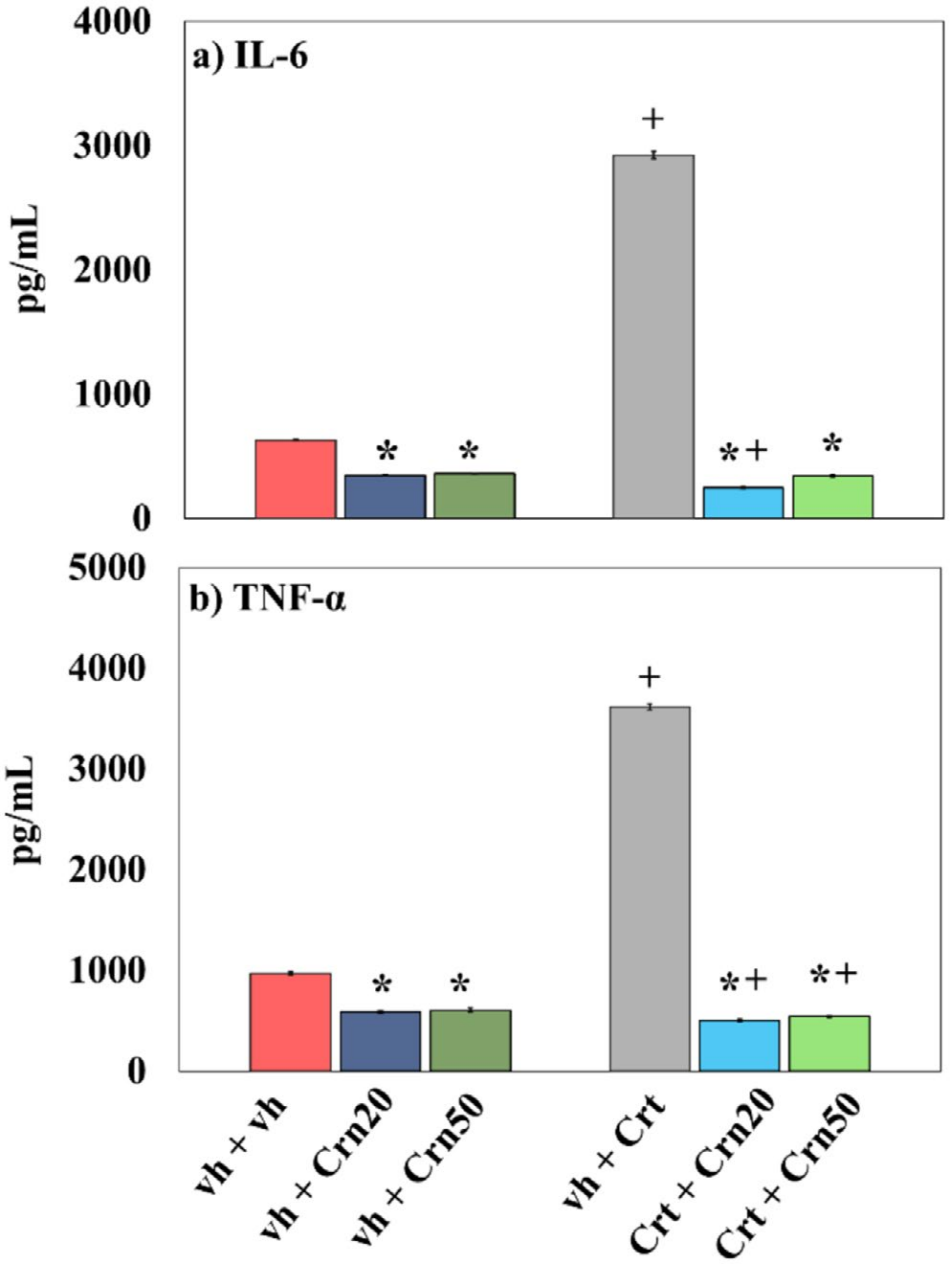
Result of Crn on cytokine levels [IL‐6 (a) and TNF‐α (b)] in vh‐, Crn‐, and Crt‐administered rats. Results are given as mean ± SD (*n* = 6). The statistics were subjected to two‐way Anova for statistical analysis followed by Tukey's multiple comparison test. Statistical significance is designated as *p < 0.05 compared with vh + vh group; +*p* < 0.05 compared with vh + Crt group.

The result of Crn shows the levels of cytokines in the hippocampal region of Crt‐treated animals in Figure 5. Data analyzed by two‐way Anova proved extensive response of Crn {(*F*_2,30_ = 54772.09; *p* = 0.000), Crt (*F*_1,30_ = 31310.90; *p* = 0.000), and correlation Crn × Crt (*F*_2,30_ = 35,764; *p* = 0.000)}. The results of Post hoc test revealed that Crt rats had elevated IL‐6 in the hippocampus. Intervention of Crn at both doses reduced IL‐6 levels in Crt‐injected groups.

Data regarding TNF‐α levels were identified by two‐way Anova demonstrating evident alteration of Crn {(*F*_2,30_ = 33538.09; *p* = 0.000), Crt (*F*_1,30_ = 17497.85; *p* = 0.000), and correlation Crn × Crt (*F*_2,30_ = 20685.73; *p* = 0.000)}. The result of Tukey's test displayed that Crt animals had elevated TNF‐α in the hippocampus. Intervention of Crn at both doses decreased TNF‐α levels in vh + Crt and Crt + Crn‐treated rats.

### 3.5 Antioxidant Enzymes

**FIGURE 6:**
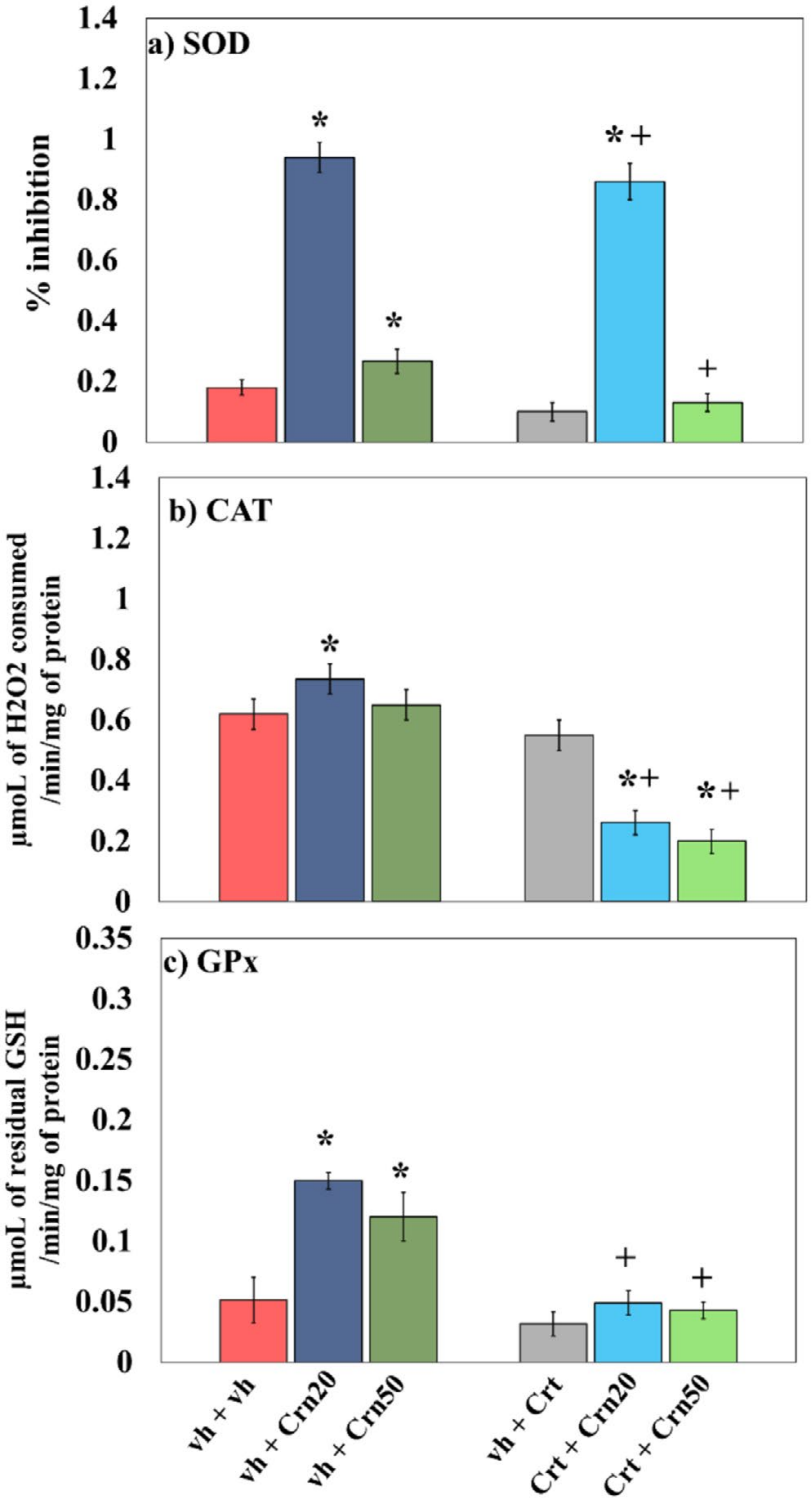
The effects of Crn on the hippocampal SOD (a), CAT (b), and GPx (c) in vh‐, Crn‐, and Crt‐administered rats. Values are expressed as mean ± SD (*n* = 6). All statistical assessments were conducted via two‐way Anova by two‐way Anova followed by Tukey's post hoc test, that is, *p < 0.05 compared to vh + vh group; +*p* < 0.05 compared to vh + Crt group.

Figure 6 illustrates the influence of Crn on enzymatic antioxidants' activity in the rat hippocampus (i.e., SOD, CAT GPx). Data analyses were carried out using 2‐way Anova.

Results concerning SOD activity displayed significant impact of Crn (*F*_2,30_ = 1025.87; *p* = 0.000), Crt (*F*_1,30_ = 42.688; *p* = 0.000), while insignificant effect in combination Crn × Crt (*F*_2,30_ = 1.188; *p* = 0.319). Tukey's multiple comparison test showed that following Crt administration, a marked decline in SOD % inhibition was detected. However, both doses of Crn improved the % inhibition by SOD and expressed improved activity of SOD in Crt and vh‐treated rats.

Findings related to CAT exhibited extensive influence of Crn (*F*_2,30_ = 31.796; *p* = 0.000), Crt (*F*_1,30_ = 409.869; *p* = 0.000) and combination Crn × Crt (*F*_2,30_ = 64.014; *p* = 0.000). Post hoc comparison using Tuckey's method showed that Crt treatment diminished CAT activity. On the other hand, both doses of Crn increased CAT in vh + Crt and Crt + Crn‐administered rats.

Analysis of Gpx parameters presented a notable difference of Crn (*F*_2,30_ = 19.608; *p* = 0.000), Crt (*F*_2,30_ = 67.688; *p* = 0.000), and the combination Crn × Crt (*F*_2,30_ = 10.297; *p* = 0.000). Tukey's post Hoc demonstrated that Crt intervention decreased GPx activity. Conversely, both doses of Crn increased GPx in vh + Crt and Crt + Crn‐injected rats.

### 3.6 5‐HT and 5‐HIAA

**FIGURE 7:**
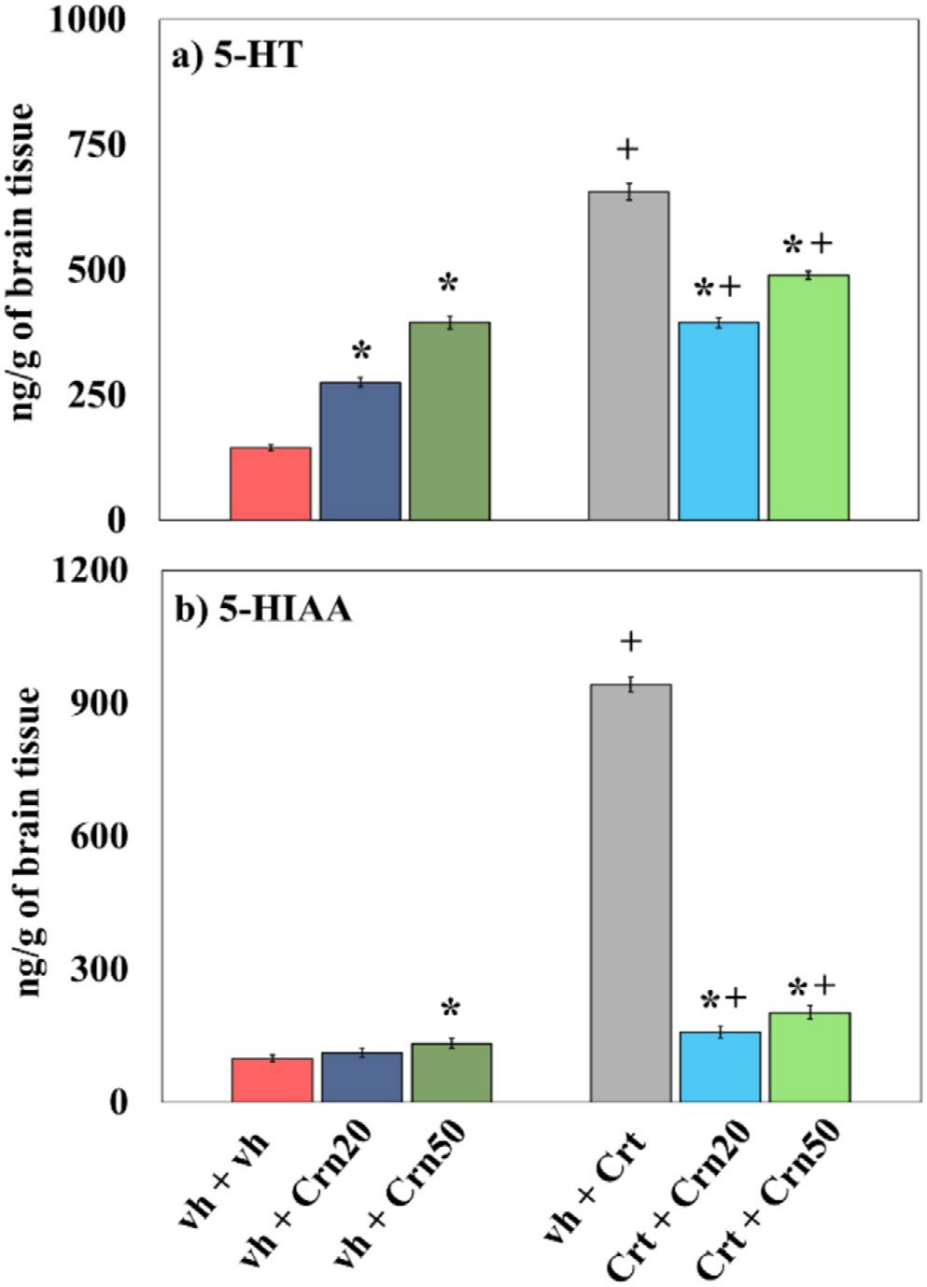
Impact of Crn on (a) 5‐HT (b) 5‐HIAA in vh and Crt‐treated rats. The values are demonstrated as mean ± SD (*n* = 6). The data set was analyzed using two‐way Anova and Tukey's multiple comparison test. Significance between groups is represented as *p < 0.05 vh + vh group and + *p* < 0.05 versus vh + Crt‐treated animals.

Figure 7 shows levels of serotonin in the rat hippocampal tissue treated with Crn and Crt. Statistical evaluation using two‐way Anova demonstrated substantial response of Crn (*F*_2,30_ = 295.9; *p* = 0.000), Crt (*F*_1,30_ = 4459.91; *p* = 0.000), and correlation Crn × Crt (*F*_2,30_ = 1404.02; *p* = 0.000). Statistical analysis Tukey' method showed that Crt exposure raised 5‐HT levels within hippocampal region of rats. Dosing with Crn increased 5‐HT levels in vh‐treated animals while reduced 5‐HT levels in Crt‐treated animals. The levels of 5‐HT were greater in animals receiving Crt + Crn injections compared to vh + Crn group animals.

Data on the activity of 5‐HIAA was assessed by 2‐way Anova, demonstrating substantial response of Crn (*F*_2,30_ = 3270.56; *p* = 0.000), Crt (*F*_1,30_ = 5490.19; *p* = 0.000), and correlation Crn × Crt (*F*_2,30_ = 3672.77; *p* = 0.000). Tukey's test showed that 5‐HIAA levels increased in rats receiving Crt. Administration of Crn increased the levels of 5‐HIAA in vh‐treated animals (at Crn50), while decreased in Crt‐treated animals at both doses. The levels of 5‐HIAA were higher in Crt + Crn‐treated in comparison with animals treated with vh + Crn.

### 3.7 AChE

**FIGURE 8:**
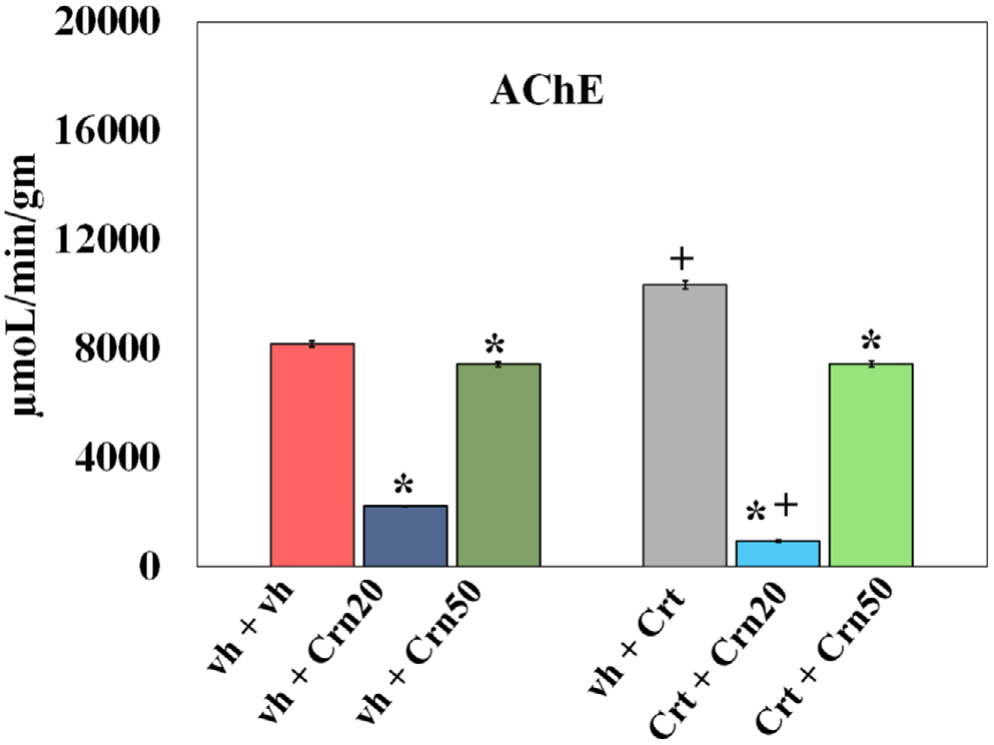
The results of Crn treatment on AChE activity in vh and Crt administered rats. Each value represents the mean ± SD (*n* = 6). Two‐way Anova was used to investigate the data, with Tukey' post hoc test for multiple comparisons. Statistical difference is represented as *p < 0.05 compared to vh + vh group and + *p* < 0.05 compared to vh + Crt‐treated animals. Dosing at both concentrations of Crn resulted in decreased AChE activity in vh and Crt‐injected groups.

Figure 8 illustrates the modulatory results of Crn on AChE enzyme function in the rat brain. The 2‐way Anova revealed a significant impact of Crn (*F*_2,30_ = 20,635.2; *p* = 0.000) and Crt (*F*_1,30_ = 88.573; *p* = 0.000), and their combination (*F*_2,30_ = 974.284; *p* = 0.000). Tukey's post hoc test exhibited that Crt administration increased AChE activity when compared with vh controls. Conversely, Crn treatment resulted in lowering AChE levels in both the vh + Crn and Crt + Crn groups.

### 3.8 Molecular Docking

**FIGURE 9:**
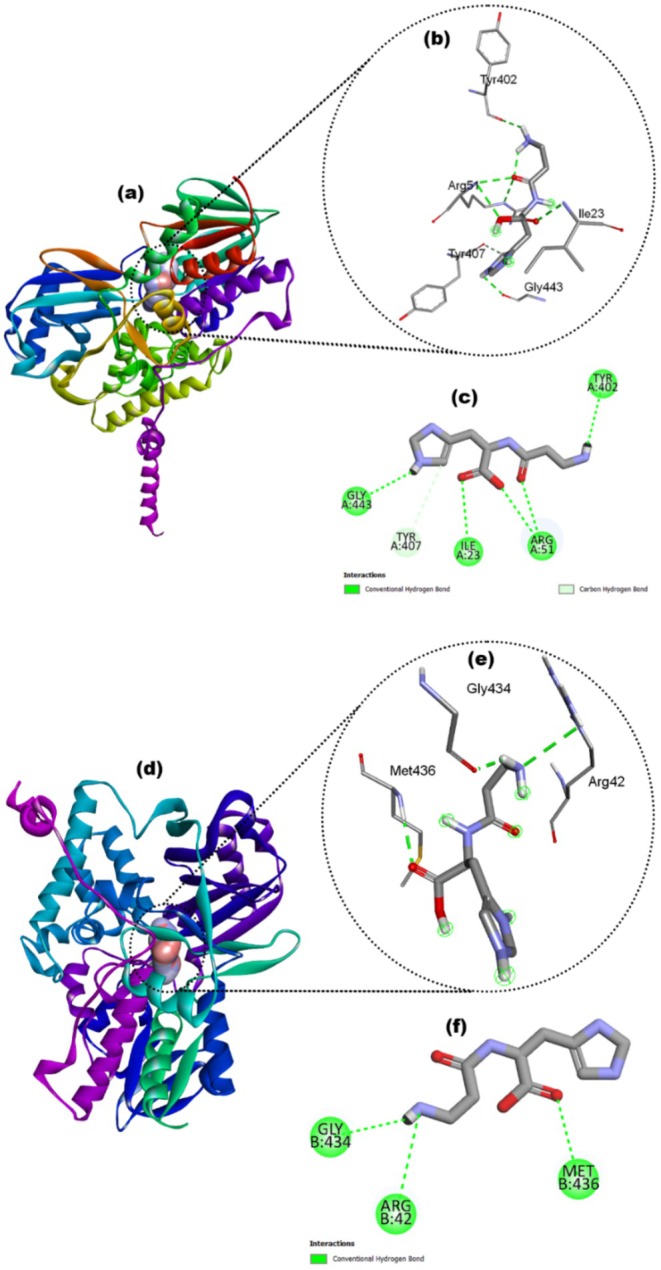
Docked complexes of Crn with MOA‐A (a–c) and MOA‐B (d–f). 3D structure of docked complex of carnosine‐MOA‐A (a), showing the interaction of Crn with the residues in 3D (b) and 2D (c). 3D structure of docked complex of carnosine‐MOA‐B (d), showing the interaction of Crn with the residues in 3D (e) and 2D (f).

Carnosine showed good binding affinity (−7.1 kcal/mol) with MAO‐A, forming hydrogen bonds with five residues: Ile23, Arg51, Tyr402, Tyr407, and Gly443, as illustrated in Figure 9a–c. Meanwhile, its binding affinity with MAO‐B was −6.7 kcal/mol, with hydrogen bonds involving only three residues: Arg42, Gly434, and Met436, as represented in Figure 9d–f.

### 3.9 High Structural Similarity Between Human and Rat MAO‐A Bound With Crn

**FIGURE 10:**
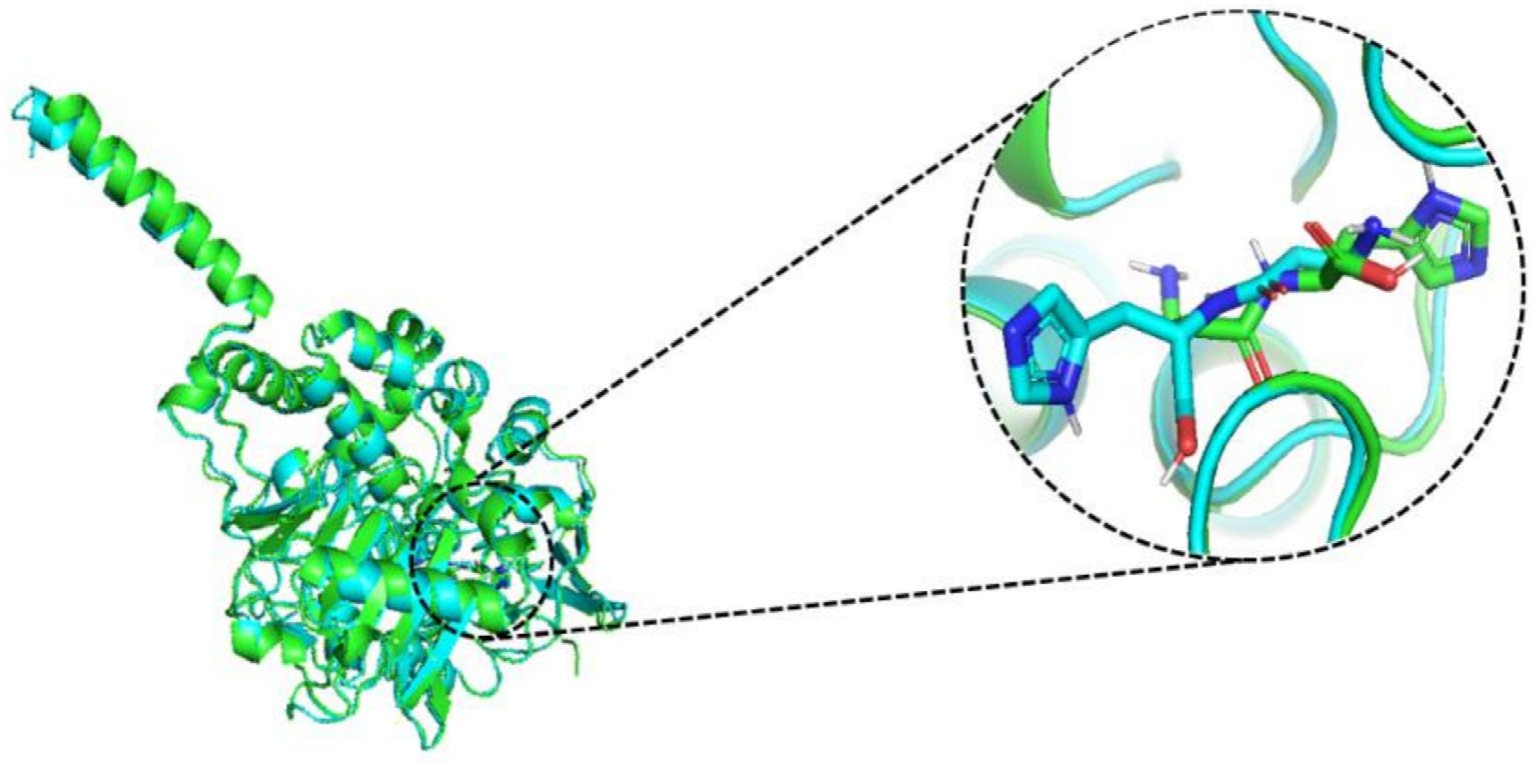
Superimposed docked complexes of the Crn molecule with human (green) and rat (cyan) MAO‐A.

We observed that the Crn molecule in both species' docked complexes occupied identical positions within the binding pocket, maintaining consistent interactions with nearby residues, as shown in Figure 10. The backbone RMSD (0.465 Å) demonstrated the close structural similarity, indicating that carnosine interacts with a conserved active site geometry in both enzymes.

### 3.10 Swiss ADME

**FIGURE 11:**
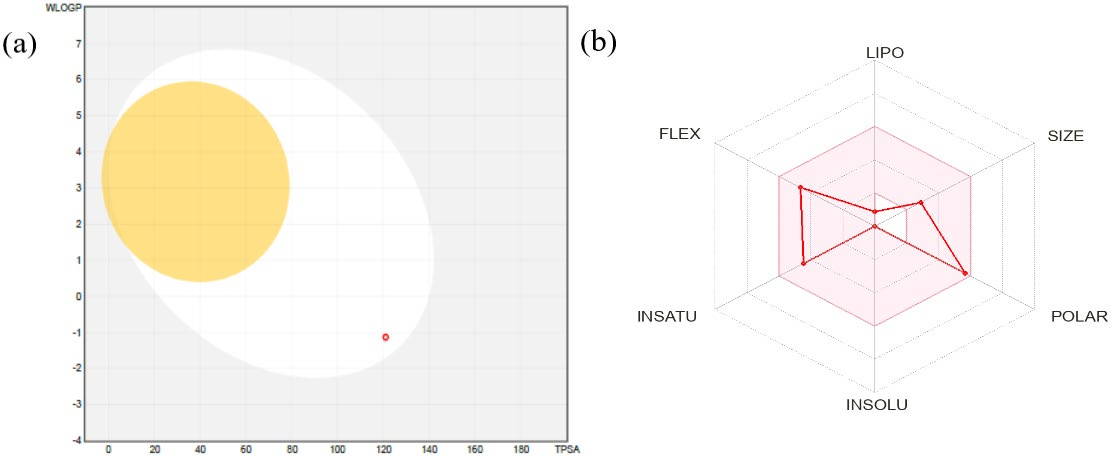
ADME profile of Crn Showing (a) Boiled egg model (b) the Radar Plot.

Crn exhibits high solubility in water and lipophilicity (−1.39). With a molecular formula of C9H14N4O3 and a molecular mass calculated as of 226.23 g/mol, it satisfies Lipinski's rule with no pharmacokinetics violations. Furthermore, Crn did not inhibit CYP2C9 or CYP2C19, confirming its potential safety. Hence, pharmacokinetic analysis indicates good bioavailability and drug‐like molecular behavior. Figure 11 shows the boiled egg model of Crn and its corresponding radar plot.

### 3.11 Histopathology

**FIGURE 12:**
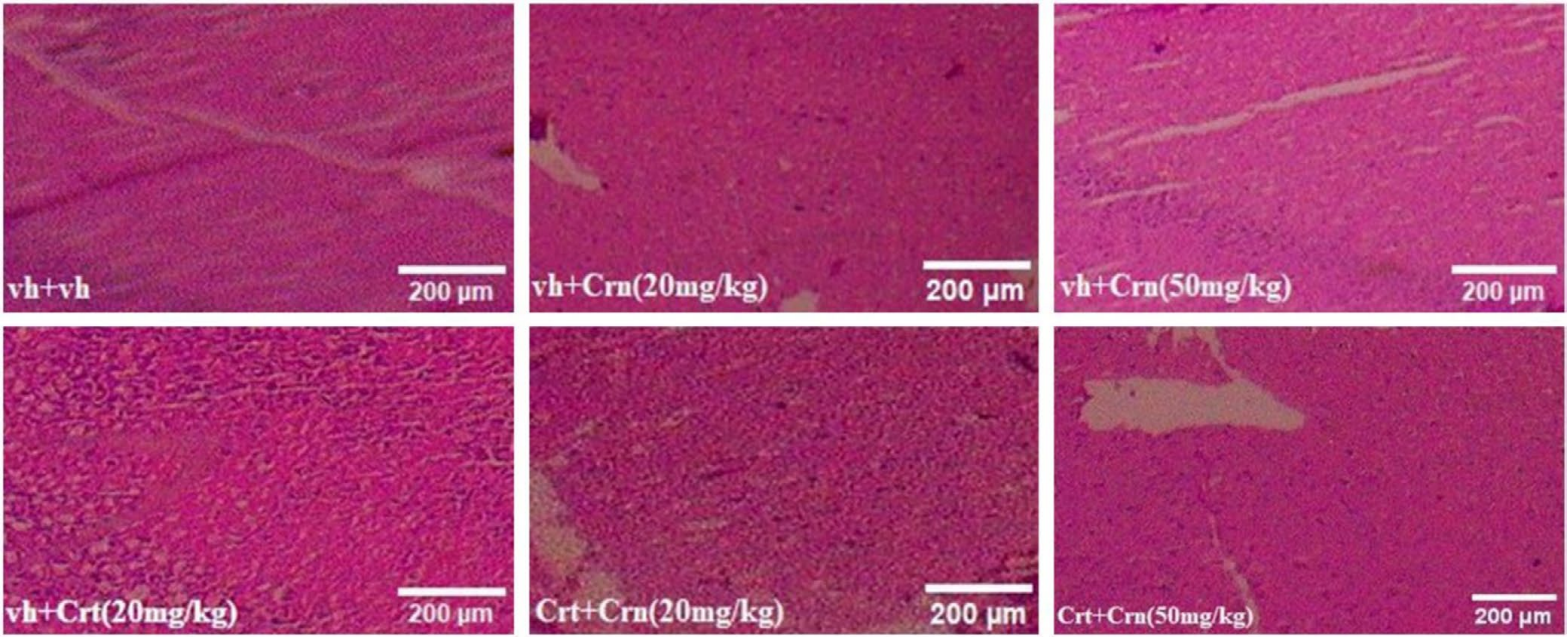
Effect of Crn on Crt induced morphological alterations in vh, Crt and Crn‐treated rats.

According to histopathological findings, the hippocampus of rats in the vh, Crt and Crn groups exhibited distinct microscopic features (Figure 12). Group (a) shows a typical nuclear membrane and neuronal arrangement. The white matter remained intact in several regions, while the nuclei exhibited a clear, round morphology, showing no evidence of pyknosis. Whereas Group (b) and Group (c) examinations show administration of both doses of Crn increased pyramidal cells while decreasing necrotic cells. The hippocampus showed no evidence of vacuolation, degeneration, or necrosis, as no darkly stained pyknotic cells were present. Group (d) displayed noticeable structural alterations in the hippocampal region, that is, cellular depletion in pyramidal and granular layers, along with darkly stained cells and noticeable vacuolation white matter region. In Groups (e) and (f), histological analysis revealed numbers of Pyramidal and granular cells were increased. Intact white matter and preserved brain tissue morphology was observed, suggesting that Crn effectively confers neuroprotection through its antioxidant effects.

## 4 Discussion

This investigation was designed to estimate the effects of Crn on Crt‐induced alterations in depressive‐like behavior, spatial memory, oxido‐neuroinflammation, antioxidant enzyme activity, serotonin metabolism, AChE activity, and histopathology in the hippocampus. Notably, coadministration of Crn attenuated Crt‐induced depressive‐like behavior (Figure 2), memory impairments (Figures 3 and 8), accompanied by disrupted serotonin metabolism (Figure 7), redox imbalance (Figures 4 and 6), neuroinflammation (Figure 5), and morphological changes in the hippocampus.

TST and MWM are well‐validated paradigms for assessing depression‐like behavior and spatial memory, respectively, in rodent models. The TST measures immobility time, a marker of behavioral despair. Previous studies have shown that acute (Li et al. 2019) and chronic (Wu et al. 2023) Crt's administration increases immobility in TST, reflecting depressive behavior. Likewise, the MWM is a widely established method for evaluating spatial learning and memory, where acute (McReynolds et al. 2014) and chronic (Trofimiuk and Braszko 2015) Crt has been shown to prolong escape latency and disrupt memory consolidation (Kim et al. 2024). Consistent with these findings, the current investigation demonstrated that Crt administration prolonged the immobility time in the TST (Figure 2) and extended escape latency in the MWM (Figure 3), suggesting depressive‐like behavior and cognitive alteration, respectively. Crn has previously been reported to possess antidepressant properties and the ability to restore memory functions (Tiwari et al. 2018). Antidepressant effect of Crn has been reported in human (Kabthymer et al. 2025) and rodents (Braga et al. 2024; Tomonaga et al. 2008). Likewise, administration of Crn improves memory in both human (Masuoka et al. 2021) and rodents (Bell et al. 2024; Ndolo et al. 2023). The present study was also in agreement that Crn administration at both 20 and 50 mg/kg significantly decreased immobility period in the TST and improved escape latency in the MWM, thereby mitigating Crt‐induced depressive behavior and cognitive impairments. These results validate earlier evidence that Crn exerts neuroprotective effects via its antioxidant potential by counteracting depressive behavior and memory deficits and extend its therapeutic potential by confirming its antidepressant and memory‐enhancing activity.

As the breakdown of ROS was caused by peroxidation of membrane lipids (Jové et al. 2020), MDA levels were used as a biomarker for cytokine activity and oxidative stress (Busch and Binder 2017). Evidence indicated that by‐products of lipid peroxidation initiate necrotic and apoptotic pathways, particularly promoting oxidative stress‐driven cell death (Sayed et al. 2022). In line with earlier reports, Crt exposure inhibited antioxidant defense and increased free radical generation (Zhao et al. 2008). Natural antioxidant enzymes with the ability to track down free radicals were SOD, CAT, and GPx (Heng et al. 2021). These enzymes function in a complementary fashion: SOD facilitates the conversion of superoxide radicals into H_2_O_2_, and CAT then reduces H_2_O_2_ to water and oxygen. In contrast, GPx further reduces H_2_O_2_ and lipid peroxides with the use of glutathione as a substrate. Together, they preserve cellular redox homeostasis and protect tissues against oxidative damage (Ighodaro and Akinloye 2018). High MDA suppresses antioxidant defenses and stimulates inflammation through the release of cytokines (Sarlaki et al. 2022). The current study established that Crt disturbed cellular homeostasis by increasing MDA and lipid peroxidation and decreasing activity of enzymatic antioxidants (SOD, CAT, GPx) and levels of pro‐inflammatory cytokines (TNF‐α, IL‐6). In comparison, both doses (20 and 50 mg/kg) of Crn restored Crt‐induced behavioral abnormalities by enhancing SOD, CAT, and GPx activities while reducing MDA levels. These findings suggest that Crn alleviates oxidative stress (Figure 4) elicited by Crt‐induced adverse effects (Decker et al. 2001). Increased hippocampal MDA (Figure 4) was associated with reduced antioxidant enzyme activity (Figure 6), which confirms that Crt‐generated oxidative stress may initiate neuronal apoptosis via inflammation and cytokine‐mediated mechanisms (Shini and Kaiser 2009). According to earlier research, Crt administrations in rats resulted in increased inflammatory cytokines levels, including IL‐6 and TNF‐α levels in their hippocampal regions due to an impaired antioxidant defense system (Kelly et al. 2018). Previously, Crn has been found to stabilize mitochondrial membranes against free radical damage, diminish lipid peroxidation, and break down advanced glycation end products (Taskin 2014). It was suggested that the antioxidant, antidepressant, and memory‐stimulating actions of Crn can be explained by the ability of L‐histidine and its imidazole ring to exist (Boldyrev et al. 2013). Singlet oxygen can react with imidazole compounds to form endoperoxide products. It was, therefore, observed that Crn reacted with excited state oxygen 2–4 times quicker than free L‐histidine and about twice as fast as the NH_2_ terminal L‐histidine dipeptides studied (Hartman et al. 1990). Although the present study did not observe the chemical mechanism of Crn as reported earlier, it was obvious from the study that Crn, as a potential antioxidant, decreased neuroinflammation (Figure 6) and oxidative stress (Figure 4) by enhancing antioxidant enzymes' activity (Figure 5; Tsai et al. 2010) in rats alone and co‐treated with Crt. These findings also indicated that the antidepressant and memory‐improving effects of Crn may be attributed to its potential to reduce oxido‐neuroinflammation and enhanced antioxidant activity.

5‐HT regulates mood and behavior, and its dysregulation is closely linked to depression. In previous reports, Crt administration has been linked with the change of depression‐like behavior, mainly due to its effects on serotonergic signaling (Li et al. 2019). Relatable with these studies, our examination observed significant behavioral alterations in Crt‐treated animals, as reflected by increased immobility time in TST or depressive‐like behavior (Figure 2). Various studies have indicated that Crt significantly increases 5‐HT and its catabolic product 5‐HIAA levels in different brain regions (Korte‐Bouws et al. 1996). Additionally, it has been confirmed that serotonergic neurotransmissions are strongly associated with depressive‐like behavior (Szoke‐Kovacs et al. 2020) and memory impairments (Bostancıklıoğlu 2020). In line with these findings, we also observed a marked increase in 5‐HT and 5‐HIAA levels in the hippocampus (Figure 7) of Crt‐treated animals, which may contribute to the depressive‐like behavior (Figure 2). Evidence from rodent and human studies suggests that Crn may function as a neuromodulator of the serotonergic system and may have antidepressant properties. In one study of aged rats, Crn was found to increase brain serotonin levels and 5‐HT turnover rates by normalizing the 5‐HIAA/5‐HT ratio, suggesting that it may enhance serotonergic metabolism (Banerjee et al. 2015). Additionally, it reversed decreased levels of serotonin receptors in the hippocampus and hypothalamus of aged rats (Banerjee and Poddar 2016). In rodent models of depression induced by stress, Crn was found to have antioxidant properties and improve behavioral manifestations of depression. In humans suffering from major depressive disorder (MDD), Crn supplementation was found to improve depressive symptoms, and a recently published meta‐analysis of clinical trials of Crn supplementation was consistent with these findings and found that it decreased depressive scores in patients with MDD (Kabthymer et al. 2025). The present study also showed that administration of Crn regulated the serotonin metabolism (Figure 7), decreased oxidoneuroinflammation (Figures 4 and 6), and enhanced antioxidant enzymatic activity (Figure 5) and produced antidepressant effect (Figure 2) in rats co‐treated with Crt. It is suggested that Crn via its antioxidant and neuro‐modulatory effects produced antidepressant effect.

ACh, an important neurotransmitter that influences cognition. Based on Hampel et al. (2018), memory function requires cholinergic activity. It has been demonstrated that increased ACh is linked with improved memory performance (Easton et al. 2020). Recurrent stress has been shown to impair memory and learning through an increase in levels of the AChE enzyme, which breaks down ACh. Previous studies revealed that repeated injection of Crt altered memory through enhancing AChE activity and reducing ACh (Savage et al. 2012). Consequently, rats exposed to recurrent Crt typically exhibit prolonged escape latencies reflecting both compromised learning and impaired memory retention (Segal et al. 1988). The findings of the present study verify previous reports, suggesting that Crt administration, increased AChE activity in the hippocampus (Figure 8) may decrease ACh availability at cholinergic receptors, thereby impairing cognitive functions, including learning and memory in the MWM test (Figure 3). Recent findings indicate that Crn improves memory by inhibiting AChE activity, which in turn increases the availability of ACh. In a model of cognitive impairment, Crn was shown to significantly reduce hippocampal AChE activity and improve cognitive performance in the Y‐maze test in rodents (Ahshin‐Majd et al. 2016). In a model of neurotoxicity and diabetes, Crn was shown to suppress overactive AChE activity, which was associated with cognitive improvement, and indicated that Crn has a neuromodulatory effect on cognitive functions, particularly on cholinergic transmission, which improved memory functions (Ahshin‐Majd et al. 2016). Furthermore, in the current study, Crn decreased AChE activity (Figure 8) and increased latency escape in all 3 phases of the MWM test alone and Crt co‐treated animals, suggesting a neuroprotective effect of Crn in improving memory function.

The monoamine oxidases (MAO) are responsible for regulating the crucial activities of the central nervous system. MAO‐A and MAO‐B, the two types of MAO, are mitochondrial enzymes that regulate brain function by degrading serotonin, dopamine, and norepinephrine (Duncan et al. 2012). As part of the docking process, the ligand (Crn) was selected from PubChem, and proteins were downloaded from PDB. Both of them were prepared by using Auto Dock vina tools. Molecular docking was employed to assess protein‐ligand binding affinities and interactions, yielding binding energies of −7.1 and −6.7 kcal/mol for the two proteins. The 3‐D structural analysis of Crn‐MAO complexes revealed crucial insights into how the Crn aligns with active sites of MAO‐A and MAO‐B (Figure 9). Furthermore, the similar binding poses of Crn in human and rat MAO‐A proteins imply that both enzymes recognize similar active site residues of the ligand Crn, indicating a common mechanism of binding (Figure 10). According to ADME evaluation, Crn exhibited strong absorption and bioavailability in the GI system and did not inhibit major metabolic isozymes, promising desirable pharmacokinetic and drug‐like properties. The bioavailability radar evaluated several physicochemical parameters to determine drug‐like properties, visualized through a boiled egg image of Crn. The yellow region exhibited a high likelihood for brain penetration, whereas the white region showed a high potential for intestinal passive absorption (Afzal et al. 2023). The red dot indicated that Crn does not act as a protein for P‐glycoprotein (Figure 11). Therefore, Crn may be a potential compound for designing drugs aimed at treating oxidative stress‐mediated diseases and neuropsychiatric disorders.

Histopathological examinations were performed to assess the hippocampal morphology of rats. Crn administration was found to mitigate Crt‐induced morphological alterations in the hippocampus. In rats treated with Crt, histopathological analysis revealed darkly stained neurons and abundant vacuolation around hippocampal cells, indicative of oxidative damage. Degenerative changes in pyramidal cells, with disrupted morphology and an elevated number of pyknotic cells, were also evident in these rats (Sapolsky et al. 1985). In Crn‐treated rats, the nuclear membrane appeared intact and visible, with neuronal cell bodies closely arranged. In contrast with Crt rats, the Crt + Crn (20 and 50 mg/kg) administered animals showed an absence of darkly stained pyknotic cells. Neuronal cells showed no sign of pericellular vacuolization, which suggested that both doses of Crn not only mitigate behavioral and biochemical alterations but also Crt‐induced hippocampal damage.

Though the immobility time was reduced in the TST and the escape latency was enhanced in the MWM following Crn administration, these effects may be subject to influences like alterations in general motor activity or levels of anxiety. Hence, the results of the behavioral effects of Crn should be interpreted with caution, and further studies on the assessment of locomotor and anxiety levels need to be conducted to validate the antidepressant and cognitive‐enhancing effects of Crn. One of the limitations of the current study is the absence of an evaluation of the general locomotor activity. If such an evaluation had been included, it would have helped ensure that the changes in immobility time and escape latency were not related to changes in general motor function. In addition, this would have helped ensure that the changes were related only to the effects of depression and cognition. Molecular docking is a technique used to evaluate the binding potential of a compound. In this study, it was used to evaluate the binding potential of the compound Crn in its binding activity. Although the experiment for MAO inhibitory activity was not conducted in this study, the molecular docking experiment was conducted in order to provide a mechanistic insight into the experiment. The histopathological evaluation conducted in this study is descriptive in nature. Although it had its own limitations, it provided a clear picture of the experiment. Quantitative scoring systems could be employed in the histopathological evaluation in the future. The sample size of the current study was limited, and the route of administration (i.p vs. oral) used was preclinical, which may not accurately reflect clinical conditions. Rodent models also have limitations, and Future studies should include additional behavioral and physiological studies, dosing strategies, and translational models to more accurately translate the current results to potential clinical applications of Crn.

## 5 Conclusion

In conclusion, the results indicate that Crn has the ability to alleviate behavioral deficits such as depression and memory impairment, in addition to restoring antioxidant enzyme activities, normalizing oxidative stress, inflammatory cytokines, AChE activity, and normalizing serotonergic metabolism. Moreover, the in silico study revealed the potential interaction of MAO and Crn, which may be the mechanism through which the serotonergic system was modulated, although further biochemical and enzyme studies are needed to confirm this hypothesis. However, the antioxidant and neuromodulatory effects of Crn are quite significant and may be considered a promising drug in the treatment of psychiatric and cognitive dysfunction following chemical stressor, that is, Crt.

## Author Contributions

**Emilio Mateev:** resources, writing – review and editing, methodology, investigation, formal analysis, software. **Natasha Manzoor:** writing – review and editing, writing – original draft, methodology, validation, investigation, formal analysis. **Muneezah Elahi:** writing – original draft, writing – review and editing, visualization, methodology, formal analysis, investigation. **Noreen Samad:** writing – original draft, project administration, investigation, methodology, formal analysis, conceptualization. **Yousef A. Bin Jardan:** resources, project administration, investigation, formal analysis, writing – review and editing. **Muhammad Zeeshan Ahmed:** writing – original draft, writing – review and editing, methodology, investigation, formal analysis. **Ali Irfan:** visualization, data curation, validation, funding acquisition, writing – review and editing.

## Funding

This research is funded by the Ongoing Research Funding Program (ORF‐2026‐457), King Saud University, Riyadh, Saudi Arabia.

## Ethics Statement

Institutional Bio‐ethical committee (Ref# Biochem/2025/D‐2010; Dated: 14.04.2025) was received for the animal experiment from the Department of Biochemistry, Bahauddin Zakariya University, Multan, Pakistan.

## Conflicts of Interest

The authors declare no conflicts of interest.

## Data Availability

All the data of this study are contained in this manuscript. More data related to this study can be accessed upon a reasonable request to the corresponding author or noreen.samad@bzu.edu.pk (N.S.).

## References

[fsn371837-bib-0001] Afzal, M. , A. S. Khan , B. Zeshan , et al. 2023. “Characterization of Bioactive Compounds and Novel Proteins Derived From Promising Source *Citrullus colocynthis* Along With In‐Vitro and In‐Vivo Activities.” Molecules 28: 1743. 10.3390/molecules28041743. 36838731 PMC9960351

[fsn371837-bib-0002] Ahshin‐Majd, S. , S. Zamani , T. Kiamari , Z. Kiasalari , T. Baluchnejadmojarad , and M. Roghani . 2016. “Carnosine Ameliorates Cognitive Deficits in Streptozotocin‐Induced Diabetic Rats: Possible Involved Mechanisms.” Peptides 86: 102–111. 10.1016/j.peptides.2016.10.008. 27777064

[fsn371837-bib-0003] Atsushi, T. , and H. Tamano . 2020. “New Insight Into Parkinson's Disease Pathogenesis From Reactive Oxygen Species‐Mediated Extracellular Zn2+ Influx.” Journal of Trace Elements in Medicine and Biology 61: 126545. 10.1016/j.jtemb.2020.126545. 32438294

[fsn371837-bib-0004] Banerjee, S. , T. K. Ghosh , and M. K. Poddar . 2015. “Carnosine Reverses the Aging‐Induced Down Regulation of Brain Regional Serotonergic System.” Mechanisms of Ageing and Development 152: 5–14. 10.1016/j.mad.2015.09.002. 26364584

[fsn371837-bib-0006] Banerjee, S. , and M. K. Poddar . 2015. “Carnosine: Effect on Aging‐Induced Increase in Brain Regional Monoamine Oxidase‐A Activity.” Neuroscience Research 92: 62–70. 10.1016/j.neures.2014.09.009. 25450310

[fsn371837-bib-0005] Banerjee, S. , and M. K. Poddar . 2016. “Aging‐Induced Changes in Brain Regional Serotonin Receptor Binding: Effect of Carnosine.” Neuroscience 319: 79–91. 10.1016/j.neuroscience.2016.01.032. 26808776

[fsn371837-bib-0007] Becker, J. B. , B. J. Prendergast , and J. W. Liang . 2016. “Female Rats Are Not More Variable Than Male Rats: A Meta‐Analysis of Neuroscience Studies.” Biology of Sex Differences 7: 34. 10.1186/s13293-016-0087-5. 27468347 PMC4962440

[fsn371837-bib-0008] Bell, S. M. , R. Hariharan , P. J. Laud , A. Majid , and B. De Courten . 2024. “Histidine‐Containing Dipeptide Supplementation Improves Delayed Recall: A Systematic Review and Meta‐Analysis.” Nutrition Reviews 82: 1372–1385. 10.1093/nutrit/nuad135. 38013229

[fsn371837-bib-0009] Bhatt, S. , A. N. Nagappa , and C. R. Patil . 2020. “Role of Oxidative Stress in Depression.” Drug Discovery Today 25: 1270–1276. 10.1016/j.drudis.2020.05.001. 32404275

[fsn371837-bib-0010] Birla, H. , T. Minocha , G. Kumar , A. Misra , and S. K. Singh . 2020. “Role of Oxidative Stress and Metal Toxicity in the Progression of Alzheimer's Disease.” Current Neuropharmacology 18: 552–562. 10.2174/1570159X18666200122122512. 31969104 PMC7457422

[fsn371837-bib-0011] Bocarsly, M. E. , J. R. Barson , J. M. Hauca , B. G. Hoebel , S. F. Leibowitz , and N. M. Avena . 2012. “Effects of Perinatal Exposure to Palatable Diets on Body Weight and Sensitivity to Drugs of Abuse in Rats.” Physiology & Behavior 107: 568–575. 10.1016/j.physbeh.2012.04.024. 22564493 PMC3484233

[fsn371837-bib-0012] Boldyrev, A. A. , G. Aldini , and W. Derave . 2013. “Physiology and Pathophysiology of Carnosine.” Physiological Reviews 93: 1803–1845. 10.1152/physrev.00039.2012. 24137022

[fsn371837-bib-0013] Bostancıklıoğlu, M. 2020. “Optogenetic Stimulation of Serotonin Nuclei Retrieve the Lost Memory in Alzheimer's Disease.” Journal of Cellular Physiology 235: 836–847. 10.1002/jcp.29077. 31332785

[fsn371837-bib-0014] Braga, J. D. , T. Komaru , M. Umino , et al. 2024. “Histidine‐Containing Dipeptide Deficiency Links to Hyperactivity and Depression‐Like Behaviors in Old Female Mice.” Biochemical and Biophysical Research Communications 729: 150361. 10.1016/j.bbrc.2024.150361. 38972141

[fsn371837-bib-0015] Brandão, A. A. C. , D. L. S. Deus , L. A. M. S. Duarte‐Filho , et al. 2023. “Nebulized and Intraperitoneal Ketamine Have Equivalent Antidepressant‐Like Effect in the Forced Swim and Tail Suspension Tests in Mice.” Pharmacology, Biochemistry, and Behavior 233: 173674. 10.1016/j.pbb.2023.173674. 37949377

[fsn371837-bib-0016] Busch, C. J. , and C. J. Binder . 2017. “Malondialdehyde Epitopes as Mediators of Sterile Inflammation.” Biochimica Et Biophysica Acta ‐ Molecular Cell Biology of Lipids 1862: 398–406. 10.1016/j.bbalip.2016.06.016. 27355566

[fsn371837-bib-0017] Caruso, G. , C. G. Fresta , F. Martinez‐Becerra , et al. 2017. “Carnosine Modulates Nitric Oxide in Stimulated Murine RAW 264.7 Macrophages.” Molecular and Cellular Biochemistry 431: 197–210. 10.1007/s11010-017-2991-3. 28290048 PMC5697141

[fsn371837-bib-0018] Castagné, V. , P. Moser , S. Roux , and R. D. Porsolt . 2010. “Rodent Models of Depression: Forced Swim and Tail Suspension Behavioral Despair Tests in Rats and Mice.” Current Protocols in Pharmacology 49: 5–8. 10.1002/0471141755.ph0508s49. 22294373

[fsn371837-bib-0019] Chatterjee, S. 2016. “Oxidative Stress, Inflammation, and Disease.” In Oxidative Stress and Biomaterials, 35–58. Elsevier. 10.1016/B978-0-12-803269-5.00002-4.

[fsn371837-bib-0020] Chen, Z.‐R. , J.‐B. Huang , S.‐L. Yang , and F.‐F. Hong . 2022. “Role of Cholinergic Signaling in Alzheimer's Disease.” Molecules 27: 1816. 10.3390/molecules27061816. 35335180 PMC8949236

[fsn371837-bib-0021] Chow, C. K. , and A. L. Tappel . 1972. “An Enzymatic Protective Mechanism Against Lipid Peroxidation Damage to Lungs of Ozone‐Exposed Rats.” Lipids 7: 518–524. 10.1007/BF02533017. 5055823

[fsn371837-bib-0022] Dash, U. C. , N. K. Bhol , S. K. Swain , et al. 2025. “Oxidative Stress and Inflammation in the Pathogenesis of Neurological Disorders: Mechanisms and Implications.” Acta Pharmaceutica Sinica B 15: 15–34. 10.1016/j.apsb.2024.10.004. 40041912 PMC11873663

[fsn371837-bib-0023] Decker, E. A. , V. Ivanov , B.‐Z. Zhu , and B. Frei . 2001. “Inhibition of Low‐Density Lipoprotein Oxidation by Carnosine and Histidine.” Journal of Agricultural and Food Chemistry 49: 511–516. 10.1021/jf0010533. 11305256

[fsn371837-bib-0024] Du, J. , Y. Wang , R. Hunter , et al. 2009. “Dynamic Regulation of Mitochondrial Function by Glucocorticoids.” Proceedings of the National Academy of Sciences of the United States of America 106: 3543–3548. 10.1073/pnas.0812671106. 19202080 PMC2637276

[fsn371837-bib-0025] Duman, R. S. , and G. K. Aghajanian . 2012. “Synaptic Dysfunction in Depression: Potential Therapeutic Targets.” Science 338: 68–72. 10.1126/science.1222939. 23042884 PMC4424898

[fsn371837-bib-0026] Duncan, J. , S. Johnson , and X. M. Ou . 2012. “Monoamine Oxidases in Major Depressive Disorder and Alcoholism.” Drug Discoveries &Therapeutics 6: 112–122. 10.5582/ddt.2012.v6.3.112. 22890201

[fsn371837-bib-0027] Easton, A. , M. Barros , and C. Lever . 2020. “Acetylcholine and Spontaneous Recognition Memory in Rodents and Primates.” In Behavioral Pharmacology of the Cholinergic System, Current Topics in Behavioral Neurosciences, edited by M. Shoaib and T. L. Wallace , 29–45. Springer International Publishing. 10.1007/7854_2020_132. 32462614

[fsn371837-bib-0028] Ellman, G. L. , K. D. Courtney , V. Andres , and R. M. Featherstone . 1961. “A New and Rapid Colorimetric Determination of Acetylcholinesterase Activity.” Biochemical Pharmacology 7: 88–95. 10.1016/0006-2952(61)90145-9. 13726518

[fsn371837-bib-0029] Endale, H. T. , W. Tesfaye , and T. A. Mengstie . 2023. “ROS Induced Lipid Peroxidation and Their Role in Ferroptosis.” Frontiers in Cell and Developmental Biology 11: 1226044. 10.3389/fcell.2023.1226044. 37601095 PMC10434548

[fsn371837-bib-0030] Everaert, I. , H. De Naeyer , Y. Taes , and W. Derave . 2013. “Gene Expression of Carnosine‐Related Enzymes and Transporters in Skeletal Muscle.” European Journal of Applied Physiology 113: 1169–1179. 10.1007/s00421-012-2540-4. 23124893

[fsn371837-bib-0031] Flohé, L. , and W. A. Günzler . 1984. “[12] Assays of Glutathione Peroxidase.” In Methods in Enzymology, 114–120. Elsevier. 10.1016/s0076-6879(84)05015-1 6727659

[fsn371837-bib-0032] Frank, M. G. , L. R. Watkins , and S. F. Maier . 2011. “Stress‐ and Glucocorticoid‐Induced Priming of Neuroinflammatory Responses: Potential Mechanisms of Stress‐Induced Vulnerability to Drugs of Abuse.” Brain, Behavior, and Immunity 25: S21–S28. 10.1016/j.bbi.2011.01.005. 21256955 PMC5654377

[fsn371837-bib-0033] Grases, G. , M. A. Colom , R. A. Fernandez , A. Costa‐Bauzá , and F. Grases . 2014. “Evidence of Higher Oxidative Status in Depression and Anxiety.” Oxidative Medicine and Cellular Longevity 2014: 1–5. 10.1155/2014/430216. PMC4020168 24876911

[fsn371837-bib-0034] Guliaeva, N. V. , A. B. Obidin , I. P. Levshina , A. V. Filonenko , A. M. Dupin , and A. A. Boldyrev . 1989. “[The Effect of Carnosine on Indicators of Free Radical Lipid Oxidation During Acute Stress in Rats].” Nauchnye Doklady Vyssheĭ Shkoly. Biologicheskie Nauki 5–16. 2790085

[fsn371837-bib-0035] Haider, S. , S. Khaliq , and D. J. Haleem . 2007. “Enhanced Serotonergic Neurotransmission in the Hippocampus Following Tryptophan Administration Improves Learning Acquisition and Memory Consolidation in Rats.” Pharmacological Reports 59: 53–57. 17377206

[fsn371837-bib-0036] Haider, S. , S. Khaliq , S. Tabassum , and D. J. Haleem . 2012. “Role of Somatodendritic and Postsynaptic 5‐HT1A Receptors on Learning and Memory Functions in Rats.” Neurochemical Research 37: 2161–2166. 10.1007/s11064-012-0839-5. 22814880

[fsn371837-bib-0037] Haider, S. , and S. Tabassum . 2018. “Impact of 1‐Day and 4‐Day MWM Training Techniques on Oxidative and Neurochemical Profile in Rat Brain: A Comparative Study on Learning and Memory Functions.” Neurobiology of Learning and Memory 155: 390–402. 10.1016/j.nlm.2018.09.003. 30195048

[fsn371837-bib-0038] Hampel, H. , M.‐M. Mesulam , A. C. Cuello , et al. 2018. “The Cholinergic System in the Pathophysiology and Treatment of Alzheimer's Disease.” Brain 141: 1917–1933. 10.1093/brain/awy132. 29850777 PMC6022632

[fsn371837-bib-0039] Hartman, P. E. , Z. Hartman , and K. T. Ault . 1990. “Scavenging of Singlet Molecular Oxygen by Imidazole Compounds: High and Sustained Activities of Carboxy Terminal Histidine Dipeptides and Exceptional Activity of Imidazole‐4‐Acetic Acid.” Photochemistry and Photobiology 51: 59–66. 10.1111/j.1751-1097.1990.tb01684.x. 2304979

[fsn371837-bib-0040] Heneka, M. T. , M. J. Carson , J. E. Khoury , et al. 2015. “Neuroinflammation in Alzheimer's Disease.” Lancet Neurology 14: 388–405. 10.1016/S1474-4422(15)70016-5. 25792098 PMC5909703

[fsn371837-bib-0041] Heng, N. , S. Gao , Y. Chen , et al. 2021. “Dietary Supplementation With Natural Astaxanthin From *Haematococcus pluvialis* Improves Antioxidant Enzyme Activity, Free Radical Scavenging Ability, and Gene Expression of Antioxidant Enzymes in Laying Hens.” Poultry Science 100: 101045. 10.1016/j.psj.2021.101045. PMC8005829 33752070

[fsn371837-bib-0042] Huang, Z. , X.‐M. Zhong , Z.‐Y. Li , C.‐R. Feng , A.‐J. Pan , and Q.‐Q. Mao . 2011. “Curcumin Reverses Corticosterone‐Induced Depressive‐Like Behavior and Decrease in Brain BDNF Levels in Rats.” Neuroscience Letters 493: 145–148. 10.1016/j.neulet.2011.02.030. 21334417

[fsn371837-bib-0043] Ighodaro, O. M. , and O. A. Akinloye . 2018. “First Line Defence Antioxidants‐Superoxide Dismutase (SOD), Catalase (CAT) and Glutathione Peroxidase (GPX): Their Fundamental Role in the Entire Antioxidant Defence Grid.” Alexandria Journal of Medicine 54: 287–293. 10.1016/j.ajme.2017.09.001.

[fsn371837-bib-0044] Ishioh, M. , T. Nozu , S. Miyagishi , et al. 2025. “Carnosine Improves Colonic Hyperpermeability Through the Brain Histamine H1 Receptor, Basal Forebrain Cholinergic Neurons, Adenosine A2B Receptors and Vagus Nerve in Rats.” European Journal of Pharmacology 1002: 177844. 10.1016/j.ejphar.2025.177844. 40516846

[fsn371837-bib-0045] Jové, M. , N. Mota‐Martorell , I. Pradas , M. Martín‐Gari , V. Ayala , and R. Pamplona . 2020. “The Advanced Lipoxidation End‐Product Malondialdehyde‐Lysine in Aging and Longevity.” Antioxidants 9: 1132. 10.3390/antiox9111132. 33203089 PMC7696601

[fsn371837-bib-0046] Kabthymer, R. H. , S. Saadati , M. Lee , et al. 2025. “Carnosine/Histidine‐Containing Dipeptide Supplementation Improves Depression and Quality of Life: Systematic Review and Meta‐Analysis of Randomized Controlled Trials.” Nutrition Reviews 83: e54–e64. 10.1093/nutrit/nuae021. 38545720 PMC12013809

[fsn371837-bib-0047] Kelly, K. A. , L. T. Michalovicz , J. V. Miller , V. Castranova , D. B. Miller , and J. P. O'Callaghan . 2018. “Prior Exposure to Corticosterone Markedly Enhances and Prolongs the Neuroinflammatory Response to Systemic Challenge With LPS.” PLoS One 13: e0190546. 10.1371/journal.pone.0190546. 29304053 PMC5755880

[fsn371837-bib-0048] Kim, H. , E. Ko , O. Kwon , and S. Jung . 2024. “Prenatal Treatment With Corticosterone via Maternal Injection Induces Learning and Memory Impairments via Delaying Postsynaptic Development in Hippocampal ca1 Neurons of Rats.” Journal of Neuroscience Research 102: e25323. 10.1002/jnr.25323. 38553948

[fsn371837-bib-0049] Korte‐Bouws, G. A. , S. M. Korte , E. R. De Kloet , and B. Bohus . 1996. “Blockade of Corticosterone Synthesis Reduces Serotonin Turnover in the Dorsal Hippocampus of the Rat as Measured by Microdialysis.” Journal of Neuroendocrinology 8: 877–881. 8933365 10.1046/j.1365-2826.1996.05389.x

[fsn371837-bib-0050] Krishnan, V. , and E. J. Nestler . 2008. “The Molecular Neurobiology of Depression.” Nature 455: 894–902. 10.1038/nature07455. 18923511 PMC2721780

[fsn371837-bib-0051] Li, Y. , Y. Zu , X. Li , et al. 2019. “Acute Corticosterone Treatment Elicits Antidepressant‐Like Actions on the Hippocampal 5‐HT and the Immobility Phenotype.” Brain Research 1714: 166–173. 10.1016/j.brainres.2019.02.022. 30794767

[fsn371837-bib-0052] Lim, D. W. , J. Park , J. Jung , et al. 2020. “Dicaffeoylquinic Acids Alleviate Memory Loss via Reduction of Oxidative Stress in Stress‐Hormone‐Induced Depressive Mice.” Pharmacological Research 161: 105252. 10.1016/j.phrs.2020.105252. 33086080

[fsn371837-bib-0053] Lin, M. T. , and M. F. Beal . 2006. “Mitochondrial Dysfunction and Oxidative Stress in Neurodegenerative Diseases.” Nature 443: 787–795. 10.1038/nature05292. 17051205

[fsn371837-bib-0054] Mahar, I. , F. R. Bambico , N. Mechawar , and J. N. Nobrega . 2014. “Stress, Serotonin, and Hippocampal Neurogenesis in Relation to Depression and Antidepressant Effects.” Neuroscience and Biobehavioral Reviews 38: 173–192. 10.1016/j.neubiorev.2013.11.009. 24300695

[fsn371837-bib-0055] Masuoka, N. , C. Lei , H. Li , and T. Hisatsune . 2021. “Influence of Imidazole‐Dipeptides on Cognitive Status and Preservation in Elders: A Narrative Review.” Nutrients 13: 397. 10.3390/nu13020397. 33513893 PMC7912684

[fsn371837-bib-0056] Masuoka, N. , C. Yoshimine , M. Hori , et al. 2019. “Effects of Anserine/Carnosine Supplementation on Mild Cognitive Impairment With APOE4.” Nutrients 11: 1626. 10.3390/nu11071626. 31319510 PMC6683059

[fsn371837-bib-0057] McEwen, B. S. 2007. “Physiology and Neurobiology of Stress and Adaptation: Central Role of the Brain.” Physiological Reviews 87: 873–904. 10.1152/physrev.00041.2006. 17615391

[fsn371837-bib-0058] McReynolds, J. R. , C. M. Holloway‐Erickson , T. U. Parmar , and C. K. McIntyre . 2014. “Corticosterone‐Induced Enhancement of Memory and Synaptic Arc Protein in the Medial Prefrontal Cortex.” Neurobiology of Learning and Memory 112: 148–157. 10.1016/j.nlm.2014.02.007. 24603007 PMC4517463

[fsn371837-bib-0059] Meftahi, G. H. , and G. P. Jahromi . 2023. “Biochemical Mechanisms of Beneficial Effects of Beta‐Alanine Supplements on Cognition.” Biochemistry (Moscow) 88: 1181–1190. 10.1134/S0006297923080114. 37758316

[fsn371837-bib-0060] Mirzakhani, N. , A. A. Farshid , E. Tamaddonfard , M. Imani , A. Erfanparast , and F. Noroozinia . 2018. “Carnosine Improves Functional Recovery and Structural Regeneration After Sciatic Nerve Crush Injury in Rats.” Life Sciences 215: 22–30. 10.1016/j.lfs.2018.10.043. 30391465

[fsn371837-bib-0061] Munhoz, C. D. , L. B. Lepsch , E. M. Kawamoto , et al. 2006. “Chronic Unpredictable Stress Exacerbates Lipopolysaccharide‐Induced Activation of Nuclear Factor‐κB in the Frontal Cortex and Hippocampus via Glucocorticoid Secretion.” Journal of Neuroscience 26: 3813–3820. 10.1523/JNEUROSCI.4398-05.2006. 16597735 PMC6674142

[fsn371837-bib-0062] Naskar, S. , A. Islam , U. K. Mazumder , P. Saha , P. K. Haldar , and M. Gupta . 2009. “In Vitro and In Vivo Antioxidant Potential of Hydromethanolic Extract of *Phoenix dactylifera* Fruits.” Journal of Scientific Research 2: 144–157. 10.3329/jsr.v2i1.2643.

[fsn371837-bib-0063] Ndolo, R. O. , L. Yu , Y. Zhao , et al. 2023. “Carnosine‐Based Reversal of Diabetes‐Associated Cognitive Decline via Activation of the Akt/mTOR Pathway and Modulation of Autophagy in a Rat Model of Type 2 Diabetes Mellitus.” Dementia and Geriatric Cognitive Disorders 52: 156–168. 10.1159/000530605. 37075707

[fsn371837-bib-0064] Ommati, M. M. , R. Heidari , V. Ghanbarinejad , A. Aminian , N. Abdoli , and H. Niknahad . 2020. “The Neuroprotective Properties of Carnosine in a Mouse Model of Manganism Is Mediated via Mitochondria Regulating and Antioxidative Mechanisms.” Nutritional Neuroscience 23: 731–743. 10.1080/1028415X.2018.1552399. 30856059

[fsn371837-bib-0065] O'Toole, T. E. , A. R. Amraotkar , H. Gao , et al. 2025. “Carnosine Supplementation Improves Cognitive Outcomes in Younger Participants of the NEAT Trial.” Neurotherapeutics 22: e00541. 10.1016/j.neurot.2025.e00541. 39919936 PMC12014415

[fsn371837-bib-0066] Pari, L. , and M. Latha . 2004. “Protective Role of *Scoparia dulcis* Plant Extract on Brain Antioxidant Status and Lipidperoxidation in STZ Diabetic Male Wistar Rats.” BMC Complementary and Alternative Medicine 4: 16. 10.1186/1472-6882-4-16. 15522116 PMC533881

[fsn371837-bib-0067] Pérez‐Nievas, B. G. , B. García‐Bueno , J. R. Caso , L. Menchén , and J. C. Leza . 2007. “Corticosterone as a Marker of Susceptibility to Oxidative/Nitrosative Cerebral Damage After Stress Exposure in Rats.” Psychoneuroendocrinology 32: 703–711. 10.1016/j.psyneuen.2007.04.011. 17561353

[fsn371837-bib-0068] Radley, J. J. , A. B. Rocher , M. Miller , et al. 2006. “Repeated Stress Induces Dendritic Spine Loss in the Rat Medial Prefrontal Cortex.” Cerebral Cortex 16: 313–320. 10.1093/cercor/bhi104. 15901656

[fsn371837-bib-0069] Samad, N. , A. Imran , S. A. Bhatti , et al. 2022. “Vitamin D2 Protects Acute and Repeated Noise Stress Induced Behavioral, Biochemical, and Histopathological Alterations: Possible Antioxidant Effect.” Saudi Journal of Biological Sciences 29: 601–609. 10.1016/j.sjbs.2021.09.018. 35002456 PMC8716964

[fsn371837-bib-0070] Samad, N. , S. Jabeen , I. Imran , I. Zulfiqar , and K. Bilal . 2019. “Protective Effect of Gallic Acid Against Arsenic‐Induced Anxiety−/Depression‐ Like Behaviors and Memory Impairment in Male Rats.” Metabolic Brain Disease 34: 1091–1102. 10.1007/s11011-019-00432-1. 31119507

[fsn371837-bib-0071] Samad, N. , N. Manzoor , Z. Muneer , S. A. Bhatti , and I. Imran . 2021. “Reserpine‐Induced Altered Neuro‐Behavioral, Biochemical and Histopathological Assessments Prevent by Enhanced Antioxidant Defence System of Thymoquinone in Mice.” Metabolic Brain Disease 36: 2535–2552. 10.1007/s11011-021-00789-2. 34309746

[fsn371837-bib-0072] Sánchez‐Martínez, C. , L. Torres‐González , G. Alarcón‐Galván , et al. 2020. “Anti‐Inflammatory and Antioxidant Activity of Essential Amino Acid α‐Ketoacid Analogues Against Renal Ischemia–Reperfusion Damage in Wistar Rats.” Biomédica 40: 336–348. 10.7705/biomedica.4875. 32673461 PMC7505519

[fsn371837-bib-0073] Sapolsky, R. , L. Krey , and B. McEwen . 1985. “Prolonged Glucocorticoid Exposure Reduces Hippocampal Neuron Number: Implications for Aging.” Journal of Neuroscience 5: 1222–1227. 10.1523/JNEUROSCI.05-05-01222.1985. 3998818 PMC6565052

[fsn371837-bib-0074] Sarlaki, F. , Z. Shahsavari , F. Goshadrou , F. Naseri , M. Keimasi , and M. Sirati‐Sabet . 2022. “The Effect of Ghrelin on Antioxidant Status in the Rat's Model of Alzheimer's Disease Induced by Amyloid‐Beta.” Biomedicine 12: 44–54. 10.37796/2211-8039.1341. 36816173 PMC9910231

[fsn371837-bib-0075] Savage, L. M. , J. M. Hall , and L. S. Resende . 2012. “Translational Rodent Models of Korsakoff Syndrome Reveal the Critical Neuroanatomical Substrates of Memory Dysfunction and Recovery.” Neuropsychology Review 22: 195–209. 10.1007/s11065-012-9194-1. 22528861 PMC5113815

[fsn371837-bib-0076] Sayed, S. , S. S. Alotaibi , A. M. El‐Shehawi , et al. 2022. “The Anti‐Inflammatory, Anti‐Apoptotic, and Antioxidant Effects of a Pomegranate‐Peel Extract Against Acrylamide‐Induced Hepatotoxicity in Rats.” Life 12: 224. 10.3390/life12020224. 35207511 PMC8878900

[fsn371837-bib-0077] Schön, M. , A. Mousa , M. Berk , et al. 2019. “The Potential of Carnosine in Brain‐Related Disorders: A Comprehensive Review of Current Evidence.” Nutrients 11: 1196. 10.3390/nu11061196. 31141890 PMC6627134

[fsn371837-bib-0078] Segal, M. , V. Greenberger , M. Israeli , and A. Biegon . 1988. “A Correlation Between Regional Acetylcholinesterase Activity in Rat Brain and Performance in a Spatial Task.” Behavioural Brain Research 30: 215–219. 10.1016/0166-4328(88)90150-7. 3166718

[fsn371837-bib-0079] Shakeel, W. , S. Javaid , S. M. M. Anjum , et al. 2020. “Time Course Evaluation of Lacosamide Alone and in Polypharmacy on Behavioral Manifestations and Oxidative Stress in Lithium‐Pilocarpine‐Induced Model.” Journal of Physiology and Pharmacology 71: 547–564. 10.26402/jpp.2020.4.10. 33316769

[fsn371837-bib-0080] Shaw, G. A. , A. J. Wegener , and G. N. Neigh . 2024. “Chronic Corticosterone Administration Alters Synaptic Mitochondrial Function Within the Hippocampus of C57Bl/6NTac Mice.” Physiology & Behavior 287: 114681. 10.1016/j.physbeh.2024.114681. 39209050 PMC12021453

[fsn371837-bib-0081] Shini, S. , and P. Kaiser . 2009. “Effects of Stress, Mimicked by Administration of Corticosterone in Drinking Water, on the Expression of Chicken Cytokine and Chemokine Genes in Lymphocytes.” Stress 12: 388–399. 10.1080/10253890802526894. 19006006

[fsn371837-bib-0082] Srinivas, U. S. , B. W. Q. Tan , B. A. Vellayappan , and A. D. Jeyasekharan . 2019. “ROS and the DNA Damage Response in Cancer.” Redox Biology 25: 101084. 10.1016/j.redox.2018.101084. 30612957 PMC6859528

[fsn371837-bib-0083] Stadtman, E. R. , and B. S. Berlett . 1998. “Reactive Oxygen‐Mediated Protein Oxidation in Aging and Disease.” Drug Metabolism Reviews 30: 225–243. 10.3109/03602539808996310. 9606602

[fsn371837-bib-0084] Sun, C. , Q. Wu , X. Zhang , Q. He , and H. Zhao . 2017. “Mechanistic Evaluation of the Protective Effect of Carnosine on Acute Lung Injury in Sepsis Rats.” Pharmacology 100: 292–300. 10.1159/000479879. 28848223

[fsn371837-bib-0085] Suvarna, K. S. , C. Layton , and J. D. Bancroft . 2018. Bancroft's Theory and Practice of Histological Techniques E‐Book. Elsevier Health Sciences.

[fsn371837-bib-0086] Swerdlow, R. H. 2018. “Mitochondria and Mitochondrial Cascades in Alzheimer's Disease.” Journal of Alzheimer's Disease 62: 1403–1416. 10.3233/JAD-170585. PMC5869994 29036828

[fsn371837-bib-0087] Szoke‐Kovacs, Z. , C. More , R. Szoke‐Kovacs , E. Mathe , and E. Frecska . 2020. “Selective Inhibition of the Serotonin Transporter in the Treatment of Depression: Sertraline, Fluoxetine and Citalopram.” Neuropsychopharmacologia Hungarica 22: 4–15. 32329748

[fsn371837-bib-0088] Tabassum, S. , S. Haider , S. Ahmad , S. Madiha , and T. Parveen . 2017. “Chronic Choline Supplementation Improves Cognitive and Motor Performance via Modulating Oxidative and Neurochemical Status in Rats.” Pharmacology, Biochemistry, and Behavior 159: 90–99. 10.1016/j.pbb.2017.05.011. 28642069

[fsn371837-bib-0089] Tanti, A. , and C. Belzung . 2010. “Open Questions in Current Models of Antidepressant Action.” British Journal of Pharmacology 159: 1187–1200. 10.1111/j.1476-5381.2009.00585.x. 20132212 PMC2848923

[fsn371837-bib-0090] Taskin, E. 2014. “L‐Carnosine Alters Some Hemorheologic and Lipid Peroxidation Parameters in Nephrectomized Rats.” Medical Science Monitor 20: 399–405. 10.12659/MSM.890528. 24614724 PMC3958568

[fsn371837-bib-0091] Thingore, C. , V. Kshirsagar , and A. Juvekar . 2021. “Amelioration of Oxidative Stress and Neuroinflammation in Lipopolysaccharide‐Induced Memory Impairment Using Rosmarinic Acid in Mice.” Metabolic Brain Disease 36: 299–313. 10.1007/s11011-020-00629-9. 33068223

[fsn371837-bib-0092] Tiwari, N. , P. Bhatia , A. Kumar , A. S. Jaggi , and N. Singh . 2018. “Potential of Carnosine, a Histamine Precursor in Rat Model of Bilateral Common Carotid Artery Occlusion‐Induced Vascular Dementia.” Fundamental & Clinical Pharmacology 32: 516–531. 10.1111/fcp.12376. 29676814

[fsn371837-bib-0093] Tomonaga, S. , H. Yamane , E. Onitsuka , et al. 2008. “Carnosine‐Induced Antidepressant‐Like Activity in Rats.” Pharmacology, Biochemistry, and Behavior 89: 627–632. 10.1016/j.pbb.2008.02.021. 18377967

[fsn371837-bib-0094] Trofimiuk, E. , and J. J. Braszko . 2015. “Ciproxifan Differentially Modifies Cognitive Impairment Evoked by Chronic Stress and Chronic Corticosterone Administration in Rats.” Behavioural Brain Research 283: 145–153. 10.1016/j.bbr.2015.01.038. 25639546

[fsn371837-bib-0095] Tsai, S.‐J. , W.‐W. Kuo , W.‐H. Liu , and M.‐C. Yin . 2010. “Antioxidative and Anti‐Inflammatory Protection From Carnosine in the Striatum of MPTP‐Treated Mice.” Journal of Agricultural and Food Chemistry 58: 11510–11516. 10.1021/jf103258p. 20925384

[fsn371837-bib-0096] Vyas, A. , S. Jadhav , and S. Chattarji . 2006. “Prolonged Behavioral Stress Enhances Synaptic Connectivity in the Basolateral Amygdala.” Neuroscience 143: 387–393. 10.1016/j.neuroscience.2006.08.003. 16962717

[fsn371837-bib-0097] Wu, C. , J. He , Y. Zhu , et al. 2023. “Ultrasound Neuromodulation Ameliorates Chronic Corticosterone‐Induced Depression‐ and Anxiety‐Like Behaviors in Mice.” Journal of Neural Engineering 20: 36037. 10.1088/1741-2552/acdea9. 37321207

[fsn371837-bib-0098] Yu, X.‐D. , D. Zhang , C.‐L. Xiao , et al. 2022. “P‐Coumaric Acid Reverses Depression‐Like Behavior and Memory Deficit via Inhibiting AGE‐RAGE‐Mediated Neuroinflammation.” Cells 11: 1594. 10.3390/cells11101594. 35626632 PMC9139330

[fsn371837-bib-0099] Zhang, J. , G. Kong , J. Yang , L. Pang , and X. Li . 2025. “Pathological Mechanisms and Treatment Progression of Alzheimer's Disease.” European Journal of Medical Research 30: 625. 10.1186/s40001-025-02886-9. 40660381 PMC12261696

[fsn371837-bib-0100] Zhang, Z. , B. Sun , M. Yang , D. Li , J. Fang , and S. Zhang . 2015. “Carnosine Attenuates Early Brain Injury Through Its Antioxidative and Anti‐Apoptotic Effects in a Rat Experimental Subarachnoid Hemorrhage Model.” Cellular and Molecular Neurobiology 35: 147–157. 10.1007/s10571-014-0106-1. 25179154 PMC11486197

[fsn371837-bib-0101] Zhao, Y. , R. Ma , J. Shen , H. Su , D. Xing , and L. Du . 2008. “A Mouse Model of Depression Induced by Repeated Corticosterone Injections.” European Journal of Pharmacology 581: 113–120. 10.1016/j.ejphar.2007.12.005. 18184609

